# Monocytic Myeloid-Derived Suppressor Cells from Tumor Tissue Are a Differentiated Cell with Limited Fate Plasticity

**DOI:** 10.4049/immunohorizons.2200079

**Published:** 2022-12-01

**Authors:** Ryan D. Calvert, James C. Fleet, Pierrick G. J. Fournier, Patricia Juarez, Grant N. Burcham, Jessica M. Haverkamp, Theresa A. Guise, Timothy L. Ratliff, Bennett D. Elzey

**Affiliations:** * Department of Science and Mathematics, Tabor College, Hillsboro, KS;; † Department of Nutrition Science, University of Texas, Austin, TX;; ‡ Center for Scientific Research and Higher Education at Ensenada, Ensenada, Baja California, Mexico;; § Heeke Animal Disease Diagnostic Laboratory, College of Veterinary Medicine, Purdue University, Dubois, IN;; ¶ Department of Comparative Pathobiology, Purdue University, West Lafayette, IN;; ∥ BlueRock Therapeutics, Boston, MA;; # Division of Internal Medicine, Department of Endocrine Neoplasia and Hormonal Disorders, The University of Texas MD Anderson Cancer Center, Houston, TX;; ** Center for Cancer Research, Purdue University, West Lafayette, IN

## Abstract

Owing to ease of access and high yield, most murine myeloid-derived suppressor cell (MDSC) knowledge comes from the study of spleen-derived MDSCs rather than those isolated from the tumor. Although several studies have identified subtle differences in suppressive function between these MDSCs, a recent report demonstrated that the whole peripheral myeloid compartment poorly reflects myeloid populations found at the tumor. We confirm and extend these observations by presenting data that indicate extensive differences exist between peripheral and tumor MDSCs, suggesting that it may be inappropriate to use spleen MDSCs as surrogates for studying tumor MDSCs. Using cytospins, we observed that tumor MDSCs have undergone a morphologic shift from immature myeloid cell forms commonly seen in bone marrow (BM) and spleen MDSCs and acquired mature myeloid cell characteristics. Spleen and BM monocyte-like MDSCs (M-MDSCs) readily responded to differentiation signals for multiple myeloid cell types whereas tumor M-MDSCs had remarkably reduced cellular plasticity. At the time of isolation, M-MDSCs from BM or spleen have little to no T cell suppressive activity whereas those from the tumor possess immediate and efficient T cell suppressive function. Finally, microarray analysis revealed that the transcriptomes of tumor and spleen M-MDSCs possessed >4500 differentially expressed transcripts. We conclude that tumor M-MDSCs are more differentiated and mature, and that they are morphologically, genetically, and functionally distinct from spleen and BM M-MDSCs. These observations have important implications for the design of anti-MDSC therapies and suggest that preclinical studies using nontumor MDSCs could lead to results not applicable to tumor MDSCs.

## INTRODUCTION

Myeloid-derived suppressor cells (MDSCs) play a critical role in the inhibition of host T cell–mediated tumor cell clearance, and their presence in tumors is strongly associated with cancer progression in mice and humans (1–4). MDSCs are described as a heterogeneous population of cells consisting of monocyte-like MDSCs (M-MDSCs, CD11b^+^Ly6C^hi^Ly6G^lo^ in mice) and polymorphonuclear-like MDSCs (PMN-MDSCs, CD11b^+^Ly6C^int^ Ly6G^hi^ in mice) (5) that have a differential ability to suppress T cells in vitro and in vivo (M-MDSCs > PMN-MDSCs) (6). MDSCs are thought to develop through a two-step process consisting of a cellular expansion in the bone marrow (BM), followed by migration to peripheral lymphoid organs and to the tumor where they carry out their immunosuppressive and tumor-supporting functions (7, 8).

In 2016, a group of scientists proposed characterization standards to define MDSCs and to serve as a guide and framework for consistent communication in MDSC research (9). They agreed that the gold standard for defining MDSCs is an in vitro functional test demonstrating suppression of T cell proliferation (9). In T cell suppression tests that are ≥48 h long, both MDSCs from the periphery or the tumor site exhibit a T cell suppressive function (10, 11). We agree that suppressive function should be the MDSC-identifying gold standard. However, we have previously shown that Gr-1^+^CD11b^+^ spleen MDSCs do not possess suppressive activity at the time of isolation, nor do they express Nos2 or Arg1 (12, 13), two factors associated with T cell suppression (14–17). As a result, based on the suppressive gold standard agreed upon by others (9), freshly isolated splenic MDSCs should not technically be considered MDSCs due to their lack of suppressive function. In contrast, we have shown that spleen MDSCs acquired suppressive activity during a 48- or 72-h assay, in part due to IFN-γ signaling (12). There are also reports showing that spleen and tumor M-MDSCs have notable differences in suppressive function—these studies were assessed in 72-h in vitro assays as well as in vivo via cell adoptive transfer and genetic knockout mice. These studies show that spleen M-MDSCs suppress in an Ag-specific, but not Ag-nonspecific, manner and depend primarily on reactive oxygen species, whereas tumor can suppress using both mechanisms and rely on NO and arginase (11). Finally, spleen MDSCs can use PD-L1 to suppress T cells whereas M-MDSCs from the tumor microenvironment (TME) do not (18). Despite these significant and well-documented differences, spleen MDSCs are routinely used as a surrogate for studying tumor MDSCs.

A recent transcriptomic study defined lung tumor myeloid subpopulations in mice and humans with remarkable clarity (19). As a result, the authors were able to clearly show that circulating human myeloid cells are distinct from the myeloid cells in the TME. This is a critical conclusion, as current dogma that contends MDSCs possess a high level of cellular plasticity is based primarily on the study of nontumor MDSCs. Unfractionated MDSCs (CD11b^+^Gr-1^+^) from the BM and spleen of tumor-bearing mice are highly plastic and can differentiate into mature osteoclasts (20–22), whereas spleen M-MDSCs can differentiate into macrophages, dendritic cells (DCs), and PMN-MDSCs (10, 23–25). Despite the lack of direct evidence, the plasticity and function defined for spleen and BM MDSCs are also routinely attributed to MDSCs within the TME. As a result, MDSCs, regardless of their tissue source, are universally described as a population of immature hematopoietic cells with phenotypic, morphological, and functional heterogeneity.

Based on our observations that the suppressive capacity of freshly isolated MDSCs is an exclusive feature of tumor MDSCs (12, 13), we contend that the application of the term “MDSCs” to cells from both peripheral tissues and the tumor does not accurately describe the functional status of the cells and creates improper understanding about their biological character. Therefore, we conducted a series of experiments to directly compare the phenotype, function, and fate plasticity of M-MDSCs isolated from various tissues of tumor-bearing mice. Our findings demonstrate that tumor M-MDSCs are a markedly different cell versus peripheral M-MDSCs, even though they share the same cell surface markers. Specifically, compared with peripheral M-MDSCs, tumor M-MDSCs have a more differentiated phenotype, exhibit a more mature cytology, possess a very different transcriptome, and exclusively demonstrate immediate suppressive activity, suggesting that only M-MDSCs from the TME possess in vivo suppressive function. Thus, M-MDSCs in the periphery poorly reflect the nature of M-MDSCs in the TME. This finding is foundational to our understanding of the tumor immune microenvironment and, based on the significant differences identified, indicates caution should be used when extrapolating spleen-derived M-MDSC results to M-MDSCs in the TME.

## MATERIALS AND METHODS

### Reagents

Routine laboratory chemicals, including DNAse I, trypsin-EDTA (0.25%), and an acid phosphatase leukocyte (tartrate-resistant acid phosphatase [TRAP]) kit, were purchased from Sigma-Aldrich (St. Louis, MO). RPMI 1640, α-MEM without ribonucleosides and deoxyribonucleosides, and DMEM with 2 mM l-glutamine were purchased from Sigma-Aldrich (St. Louis, MO) or Corning (Manassas, VA). FBS was purchased from Corning (Manassas, VA) or HyClone (South Logan, UT). HEPES (1 M) and 100 mM sodium pyruvate were purchased from Mediatech (Manassas, VA). Penicillin/streptomycin (10,000 U/ml) was purchased from HyClone. Liberase TM (thermolysin medium) was purchased from Roche (Basel, Switzerland) and used at 5 mg/ml. An EdU Click-iT kit was purchased from Life Technologies (Carlsbad, CA). An RNeasy kit was purchased from Qiagen (Hilden, Germany). TRI Reagent (Zymo Research) and Direct-zol RNA MiniPrep Plus kits were purchased from Zymo Research (Irvine, CA). All Abs, TruStain FcX, and Zombie Violet were purchased from BioLegend (San Diego, CA); anti-rat IgG compensation beads were purchased from Becton Dickinson (San Jose, CA). All recombinant murine cytokines were purchased through PeproTech (Rocky Hill, NJ). Moloney murine leukemia virus reverse transcriptase, 5× first-strand buffer, BSA, deoxynucleotide triphosphate, RNasin, random hexamers, and oligo(dT) primers were purchased from Invitrogen (Carlsbad, CA). Positive and negative selection MicroBead kits for magnetic cell purification of CD8^+^ T cells and Ly6G^+^ cells were purchased from Miltenyi Biotec (San Diego, CA).

### Mouse lines and husbandry

Female and male C57BL/6J and BALB/cJ were purchased from The Jackson Laboratory (Bar Harbor, ME), and C57BL/6N mice were purchased from the Purdue Transgenic Mouse Core Facility (West Lafayette, IN). Prostate OVA-expressing mice-3 (POET-3) mice were generated and maintained as previously described (26). Rag^−/−^ OT-I mice were generated and maintained as previously described (27). Mice were housed in specific pathogen-free conditions with a 12-h light/12-h dark cycle. Mice were fed a standard chow diet (Teklad 2018) and water ad libitum. All of the mouse use described in this study was approved by the Purdue University or Indiana University School of Medicine Animal Care and Use Committees.

### Cell culture

The following cell lines were used in our studies: RM1 cells (a gift from Dr. Tim Thompson while at Baylor University), EL4 cells (a gift from Dr. Dmitry Gabrilovich), and 4T1 cells.

All cells were grown in humidified incubators with 5% CO_2_ at 37°C. EL4, 4T1, and Rag^−/−^ OT-I T cells were cultured in complete RPMI (RPMI-C; RPMI 1640 containing 10% FBS, 1 mM HEPES, 1 mM sodium pyruvate, 100 U/ml penicillin/streptomycin). EL4 culture also included 55 μM 2-ME. RM1 cells were cultured in complete DMEM (DMEM-C; DMEM containing 10% FBS, 1 mM sodium pyruvate, 1% penicillin/streptomycin). 4T1 and RM1 cells were passaged 1:10, at 70% confluency. Nonadherent EL4 cells were maintained between 1 × 10^5^ and 1 × 10^6^ cells/ml.

During some M-MDSC differentiation experiments, tumor explant supernatant (TES) was added to RPMI-C. The protocol for creating TES was provided by Dr. Dmitry Grabrilovich. Briefly, TES was derived from s.c. tumors that developed in C57BL/6J mice 14 d after injection of 5 × 10^6^ EL4 cells. Tumors were harvested, minced, and ~500 mm^3^ of tissue was cultured with RPMI 1640 (with 10% FBS, 1% HEPES, and 1% penicillin/streptomycin) for 18 h. At 18 h the supernatant was removed and purified by filtering through a 70-μm cell strainer followed by centrifugation at 200 × *g* for 5 min at 4°C. The supernatant was then transferred to a new tube and centrifuged at 13,300 × *g* for 20 min at 4°C. Finally, the supernatant was sterile filtered (0.22 μm), aliquoted, and stored at −80°C.

### Tumor formation and inflammation

Tumor cells were suspended in PBS for all injections. Concentrated cell suspensions (5 × 10^7^ cells/ml, EL4; 10^7^ cells/ml, RM1; 3 × 10^7^ cells/ml, 4T1) in PBS were kept on ice prior to injection of 100 μl of cell suspension.

To generate tumor MDSCs for the microarray analysis, RM1 tumor cells were harvested for injection during their exponential growth phase (~50% confluent) and 1 × 10^6^ cells were injected into the peritoneal cavity of C57BL/6J mice. Seven days later, tumor-derived MDSCs were harvested from ascites by serial lavage with sterile PBS. MDSCs were isolated as described below. To generate activated peripheral MDSCs from the spleen, POET-3 mice were injected with 5 × 10^6^ activated OT-I cells to induce prostate inflammation, as we have previously described (12). Six days later the mice were killed, spleens harvested, and MDSCs were isolated as described below.

To generate MDSCs for in vitro differentiation experiments, 6–10 female 6- to 8-wk-old C57BL/6 mice were injected s.c. with 5 × 10^6^ EL4 or 1 × 10^6^ RM1 cells. BALB/cJ mice were injected s.c. with 3 × 10^6^ 4T1 cells. Tumors were allowed to grow for 14 d and did not exceed 2000 mm^3^ in volume prior to harvest. Tissue and tumors were separately pooled to isolate enough cells for each experiment. MDSCs were isolated as described below.

Finally, to generate MDSCs from osteolytic BM for osteoclast differentiation experiments, intracardial inoculation of tumor cells was performed as previously described (28). Briefly, 8- to 12-wk-old female BALB/cJ mice were anesthetized and 1 × 10^5^ 4T1 cells in 100 μl of PBS were inoculated into the left ventricle during 30 s. Tumors were allowed to grow for 7–10 d prior to harvest. The development of osteolytic lesions (OLs) in hindlimbs were identified prior to BM harvest from osteolytic and nonosteolytic bones using a Kubtec digital x-ray imager (Kubtec, Milford, CT).

### MDSC isolation

MDSCs were isolated from BM, spleen, solid tumors, or i.p. induced tumors. Mice were euthanized and then BM was removed from femurs and tibias by flushing the marrow cavity with RPMI-C. Spleens were physically disrupted between two frosted microscope slides in RPMI-C. Solid tumors were resected when they reached ~1000 mm^3^, minced, and then digested with 5 mg/ml Liberase TM (Roche Diagnostics, Indianapolis, IN) and 1 mg/ml DNase I (Sigma-Aldrich, St. Louis, MO) in PBS at 37°C for 1 h with shaking. In mice injected i.p. with tumor cells, 7 d after the injection the peritoneal cavity was washed with 3× 5 ml of sterile PBS to harvest MDSCs. Following dissociation, digestion, or collection of cells, RBCs were lysed with ACK lysis buffer (150 mM NH_4_Cl, 10 mM KHCO_3_, 0.1 mM Na_2_ EDTA) for 2–3 min at room temperature and washed twice in 1× PBS for 5 min at 200 × *g*. Samples were then filtered through a 70-μm cell strainer and spun at 300 × *g* for 5 min to pellet cells. Cells were then stained following blocking of Fc receptors with TruStain FcX (1:50) or 50% mouse serum in the dark at room temperature for 10 min. In some experiments Zombie Violet (1:100) was added to the TruStain FcX master mix so that dead cells could be identified and removed. Single-cell suspensions were labeled with anti-CD11b (clone M1/70), anti-Ly6G (clone 1A8), and anti-Ly6C (clone HK1.4) and sorted by flow cytometry (iCyt Reflection [Sony, Tokyo, Japan] or FACSAria II [Becton Dickinson, San Jose, CA]) into monocytic and granulocytic populations. For the cell differentiation studies (see Experimental design), the CD11b^+^ cell population was enriched in the samples by MACS (Miltenyi Biotec, Auburn, CA) prior to labeling with anti-Ly6C and Ly6G Abs and FACS analysis.

### Experimental design

#### Study 1. Cellular morphology of MDSCs.

Eight- to 12-wk-old female or male BALB/cJ or C57BL/6J mice were injected s.c. with 3 × 10^6^ 4T1 tumor cells in the flank. CD11b^+^Ly6C^hi^Ly6G^lo^ (M-MDSCs) and CD11b^+^Ly6^int^Ly6G^hi^ (PMN-MDSCs) cells from tumors, spleen, and BM of tumor-bearing mice were isolated as described below. Two hundred thousand cells per sample were loaded into CytoSep funnels (Fisher Scientific, Pittsburgh, PA) and centrifuged at 250 × *g* for 5 min to adhere cells to slides. After a minimum of 30 min of air-drying time, cells were stained with a modified Wright–Giemsa stain. Slides were assessed for the presence of mature and immature cell forms of the granulocytic and monocytic lineages in a double-blinded fashion and scored by a board-certified veterinary pathologist (G.N.B.). Immature myeloid forms, for example, myeloblasts and metamyelocytes, were identified as large cells with bluer cytoplasm and a higher nucleus-to-cytoplasm ratio. Immature granulocytic forms were identified as cells with typical band-shaped nuclei (e.g., nuclei that form a “C” shape with the ends of the nucleus being parallel to one another), or ring-shaped nuclei with blue cytoplasmic granules or bluer overall cytoplasm. Mature granulocytic forms were identified as segmented neutrophils or neutrophils with ring-shaped nuclei and clear granules. Monocyte forms were identified as cells with oval, bilobed, or reniform nuclei and variably vacuolated, clear to light blue cytoplasm. Murine monocytes can also display ring-shaped forms; these cells were distinguished from neutrophils with ring-shaped nuclei by having a larger, thicker nucleus. Macrophage forms were identified as larger cells with round to oval-shaped nuclei, abundant clear cytoplasm, and extensive cytoplasmic vacuolation. Each sample was examined for the percentage of each form within a cytospin: 0, 0–4%; 1, 5–24%; 2, 25–49%; 3, 50–74%; 4, 75–100%.

#### Study 2. Induced differentiation of M-MDSCs from BM, spleen, and tumor.

A series of experiments were conducted to determine whether M-MDSCs from BM, spleen, and solid tumors could respond to signals that promote differentiation into various mature myeloid cell populations. Replicate experiments with paired BM, spleen, and tumor samples were conducted for each study. Each biological replicate was comprised of a cell pool of 6–10 mice. Tissue M-MDSCs were cultured in RPMI-C media plus specific cytokine treatments for 3 d; the studies were also conducted in the presence or absence of 20% TES to assess whether signals from the TME changed the fate potential of the tissue M-MDSCs. For all experiments, 10^5^ cells were seeded into each well of a 96-well plate; within each replicate experiment, three wells were used for each treatment and wells were pooled at harvest for analysis. Following the 3-d treatment period, media and nonadherent cells were collected. Adherent cells were harvested with 0.25% trypsin-EDTA for 10–15 min or until the cell layer had dispersed. Adherent and nonadherent cells were pooled and prepared for flow cytometry analysis.

In study 2A we examined the conversion of M-MDSCs to PMN-MDSCs using two treatment designs. First, we followed the differentiation protocol reported previously by Youn et al. (25). For this approach, spleen- and tumor-isolated CD11b^+^ Ly6C^+^Ly6G^−^ cells were cultured in RPMI-C with 10 ng/ml GM-CSF in the presence or absence of 20% TES (*n* = 5 per group). Second, we repeated these experiments without GM-CSF to determine whether TES alone was a sufficient stimulus for this conversion (*n* = 8 per group).

In study 2B we examined the potential of Ly6C^+^Ly6G^−^ cells to differentiate into DCs (CD11c^+^). For this, isolated BM, spleen, and tumor Ly6C^+^Ly6G^−^ cells were cultured in RPMI-C with 10 ng/ml GM-CSF and 2 ng/ml IL-4 in the presence or absence of 20% TES (*n* = 3 per group).

In study 2C we examined the potential of BM-, spleen-, and tumor-isolated Ly6C^+^Ly6G^−^ cells to differentiate into macrophages (CD11b^+^F4/80^+^). Ly6C^+^Ly6G^−^ cells were cultured in RPMI-C supplemented with 25 ng/ml M-CSF in the presence or absence of 20% TES (*n* = 3 per group).

Finally, in study 2D we examined the potential to differentiate tissue M-MDSCs into osteoclasts using two different models. In the first model, BM, spleen, and tumor M-MDSCs from mice with solid 4T1 tumors were plated at 5 × 10^5^ cells/cm^2^ in α-MEM-C (10% FBS and 2 mM l-glutamine) supplemented with 50 ng/ml M-CSF and 100 ng/ml RANKL (*n* = 3 per group). Half of the media was renewed every 2 d and at 8 d the cells were processed with the TRAP kit according to the manufacturer’s instructions to quantify the number of osteoclasts, defined as TRAP^+^ multinucleated cells (i.e., cells with at least three nuclei) in each well. In the second model, mice were given intracardial injections of 4T1 cells (28). M-MDSCs were isolated from spleen of tumor-bearing mice and from the marrow of bones containing OLs or paired bones that did not have OLs (*n* = 3 per group). These tissue M-MDSCs were tested for their ability to undergo osteoclast differentiation as described above.

#### Study 3. M-MDSC T cell suppression activity.

For each experimental replicate, M-MDSCs were isolated from the BM, spleen, and solid tumors from three 4T1 tumor-bearing mice and the cells were pooled by tissue of origin. These experiments were repeated three times. Single-cell suspensions of tumors were prepared and CD11b^hi^Ly6C^hi^Ly6G^lo^ cells were isolated by FACS. M-MDSCs from each tissue were then resuspended at 1 × 10^6^ cells/ml. One hundred thousand MDSCs were plated together with either activated CD8^+^ T cells in the presence of 5 μg/ml anti-CD3 Ab and 2.5 μg/ml anti-CD28 Ab in flat-bottom 96-well plates for the 16-h assay or with unactivated CD8^+^ T cells and without the anti-CD3 or CD28 Abs for the 72-h assay. Positive control wells contained purified CD8^+^ T cells placed on a feeder layer of naive whole spleen cells.

Negative control wells contained either purified CD8^+^ T cells placed on a feeder layer of naive spleens cells but without added anti-CD3 or CD28 Abs or purified CD8^+^ T cells placed on a feeder layer of naive spleens cells without added EdU. Preactivation of CD8^+^ T cells for the 16-h assay was done using a single-cell suspension of splenocytes from normal BALB/cJ mice. RBCs were lysed with ACK lysis buffer and splenocytes from two spleens were placed in upright T25 tissue culture flasks with 10 ml of RPMI-C containing 5 μg/ml anti-CD3 and 2.5 μg/ml anti-CD28 for 24 h. Miltenyi Biotec positive T cell selection magnetic bead kits were used to obtain purified activated CD8^+^ T cells (>95% pure as verified by flow cytometry). One and one-half hours prior to the end of the 16- or 72-h suppression assay, 10 μM EdU was added to the appropriate wells. Cells were then harvested from the plates and prepared for FACS analysis (see below).

#### Study 4. Microarray analysis of spleen and tumor MDSC subtypes.

We previously showed that spleen and tumor MDSCs from mice with prostate inflammation and prostate tumors behave the same in T cell suppression assays (12). Cells were collected from the peritoneal cavity of mice with i.p. RM1 tumors and from the spleens of POET-3 mice with OT-1 cell–induced prostatic inflammation. Cells from three to five mice were used to make each sample, and four replicate samples were processed for each tissue. MDSC subtypes were isolated by flow cytometry sorting as described below. RNA was isolated from cell pellets of each tissue/MDSC subtype (*n* = 4 per group) using the RNeasy kit (Qiagen) and following the manufacturer’s instructions. Microarray analysis was conducted, and the data were analyzed as described below.

#### Study 5. In vitro differentiation of spleen M-MDSCs.

M-MDSCs were isolated from the solid tumor and spleen of EL4 tumor-bearing mice as described below. Cells from 6–10 mice were pooled to create each biological replicate, and we used four to five biological replicates for each group. Upon isolation, 2 × 10^5^ spleen or tumor M-MDSCs were placed in TRI Reagent, flash-frozen in liquid nitrogen, and stored at −80°C until all biological replicates were collected. An additional group of spleen M-MDSCs were cultured in RPMI-C alone or RPMI-C + 20% TES. After 72 h, cell suspensions were pelleted and resuspended into TRI Reagent (Zymo Research, Irvine, CA), flash-frozen in liquid nitrogen, and stored at −80°C until all biological replicates were collected. RNA was isolated using the Direct-zol RNA MiniPrep Plus kit (Zymo Research) following the manufacturer’s protocol. cDNA was created as we have previously reported (29) using a 1.5-h incubation at 37°C. Real-time PCR analysis was done as previously described (29) using 50 cycles of PCR. Premade PrimeTime primer/probe sets were purchased from Integrated DNA Technologies (Skokie, IL): Nos2 (Integrated DNA Technologies assay: Mm.PT.56a.43705194), Arg1 (Mm.PT.58. 8651372), VDR (Mm.PT.58.7050931), Ccr1 (Mm.PT.58.32053786), Slc7a11 (Mm.PT.58.29117975), Stfa2l1 (Mm.PT.58.41576651), Mpo (Mm.PT.58.5251395), Spp1 (Mm.PT.58.29117975), and Nr1d1 (Mm.PT.58.17472803). Ribosomal 18S mRNA level (Hs.PT.39a. 22214856.g) was used as a housekeeping gene. Relative expression levels were determined using the ΔΔCt method (30).

### Flow cytometry and analysis

#### T cell suppression assay.

Following coculture and EdU treatment of M-MDSCs and CD8^+^ T cells, cells were harvested from the plates and stained with Abs against CD8 and CD11b. Afterwards, cells were fixed and analyzed for EdU levels using the EdU Click-iT staining kit, following the manufacturer’s instructions (Life Science Technologies, Waltham, MA). In brief, following fixation, cells were resuspended in permeabilization buffer for 10 min and then samples were incubated with Click-iT reaction master mix for 30 min at room temperature. Samples were washed and resuspended in 150 μl of permeabilization buffer then analyzed for CD8, CD11b, and EdU on a BD LSRFortessa cell analyzer (BD Biosciences, San Jose, CA). Data were analyzed using FlowJo v.10 (Tree Star, Ashland, OR), that is, selection of non-debris events, gating for single cells, and biplots for cytotoxic T cells (CD11b^−^CD8^+^ cells) and for proliferating cells (side scatter area × EdU^+^). CD8^+^ T cells incubated without EdU were used to create the negative EdU gate.

#### Cell differentiation studies.

Initially Fc receptors were blocked with TruStain FcX (1:50) or 50% mouse serum, in the dark at room temperature for 10 min. In some experiments Zombie Violet (1:100) was added to the TruStain FcX master mix. A mixture consisting of fluorescent-labeled Abs against CD11b, Ly6C, Ly6G, F4/80, and CD11c was added to the cells followed by incubation for 20 min at 4°C in the dark. The negative control staining samples for F4/80 and CD11c expression was the inclusion of all fluorescent Abs with fluorescent isotype controls matched for F4/80 and CD11c (i.e., CD11b, Ly6C, Ly6G, rat IgG2a, and rat IgG-2b). After staining, cells were fixed in the dark with 10% neutral buffered formalin for 15 min at 4°C. Fixed preculture controls (Supplemental Fig. 1) and samples after 3 d of culture, as well as single-color compensation samples containing compensation beads (BD Biosciences, San Jose, CA), were analyzed by FACS on a BD LSRFortessa cell analyzer. Data were analyzed using FlowJo v.10. Prior to setting final gates, files were roughly gated to verify that the compensation matrix from the initial analysis displayed good separation of populations. When the compensation matrix resulted in spectral overlap, a new matrix was made within FlowJo using single-color controls. Following compensation verification, live-singlet CD11b^+^ cells were gated based on location of positive populations (Supplemental Fig. 1). For final M and G gates, M-MDSCs were gated as Ly6C^hi^Ly6G^−^ and PMN-MDSCs as Ly6C^int^Ly6G^hi^ based on a preculture controls that were fixed and analyzed on a BD LSRFortessa. In addition to the M-MDSC and PMN-MDSC gates, a histogram was used to quantify the total percentage of Ly6G^hi^ cells. Finally, to identify macrophages and DCs, we used isotype controls to locate the F4/80 and CD11c negative population using a quadrant gate. All gates were confirmed across samples to ensure true population separation.

### Microarray analysis and bioinformatics

Transcript levels in each sample were determined using the Affymetrix mouse gene 1.0 ST v1 GeneChip (Thermo Fisher Scientific, Waltham MA; 27,543 probe sets). RNA labeling, chip hybridization, and chip scanning were carried out at the Purdue Genomics Facility using standard Affymetrix protocols. Chips were scanned, and raw data were saved into CEL files. Microarray data and CEL files can be accessed at the National Center for Biotechnology Information Gene Expression Omnibus (https://www.ncbi.nlm.nih.gov/geo/query/acc.cgi?acc=GSE116596).

The sample array files were examined for quality and robust multiarray average normalized using RMAExpress (htttps://rmaexpress.bmbolstad.com) (quartile normalization, gene level analysis, normalized unscaled standard errors, and relative log expression plots). Arrays for all of the samples met the quality control criteria and were used in downstream analysis. Probe sets were annotated to genes using BRB-ArrayTools v4.6.0 Beta_1 (https://brb.nci.nih.gov/BRB-ArrayTools/index.html). Prior to statistical analysis, the genes in the bottom 25% of expression were removed, leaving 22,390 probe sets. Differential gene expression was conducted using significance analysis for microarrays (31) within BRB-ArrayTools (500 permutations, 5% false discover rate [FDR], 70% false-negative detection rate). Statistical analysis was conducted on four pairwise comparisons, that is, spleen granulocyte-like MDSCs (G-MDSCs) versus M-MDSCs, tumor G-MDSCs versus M-MDSCs, spleen PMN-MDSCs versus tumor PMN-MDSCs, and spleen M-MDSCs versus tumor M-MDSCs, between the four sample groups. Bioinformatics analysis was conducted using MetaCore (Clarivate, Philadelphia, PA). For each relevant two-way comparison, analysis was conducted for the combined upregulated and downregulated genes as well as separately for upregulated or downregulated genes only.

### Statistical analysis

Statistical analyses were conducted using SAS Enterprise Guide v5.1 (SAS Institute, Cary, NC). Evaluation of histograms and the Shapiro–Wilk test of normality (*p* < 0.05) were used to assess whether the data were normally distributed. When data from flow cytometry were not normally distributed, they were transformed using cube-root, natural log, or 2 arcsin $\sqrt{x/100}$ (32) transformation. After confirming the distribution, data were assessed for outliers using Cook’s *D* statistic and Studentized residuals by leverage plot, and outliers were removed. Although statistical tests were conducted on transformed data, data are reported as the mean ± SEM of nontransformed data. Tests of significance were determined for T cell suppression tests and the comparison of TRAP^+^ cells among sites by one-way ANOVA. The examination of tissue M-MDSCs for their response to differentiating agents in the presence or absence of TES was conducted using a split-plot design to account for the interdependence of responses within each replicate. For all analyses, comparisons among multiple treatment groups were conducted using Tukey’s honestly significant difference test. A *p* value of <0.05 was considered statistically significant.

## RESULTS

### Cytologic examination of M-MDSCs from BM, spleen, and tumor

**FIGURE 1.**
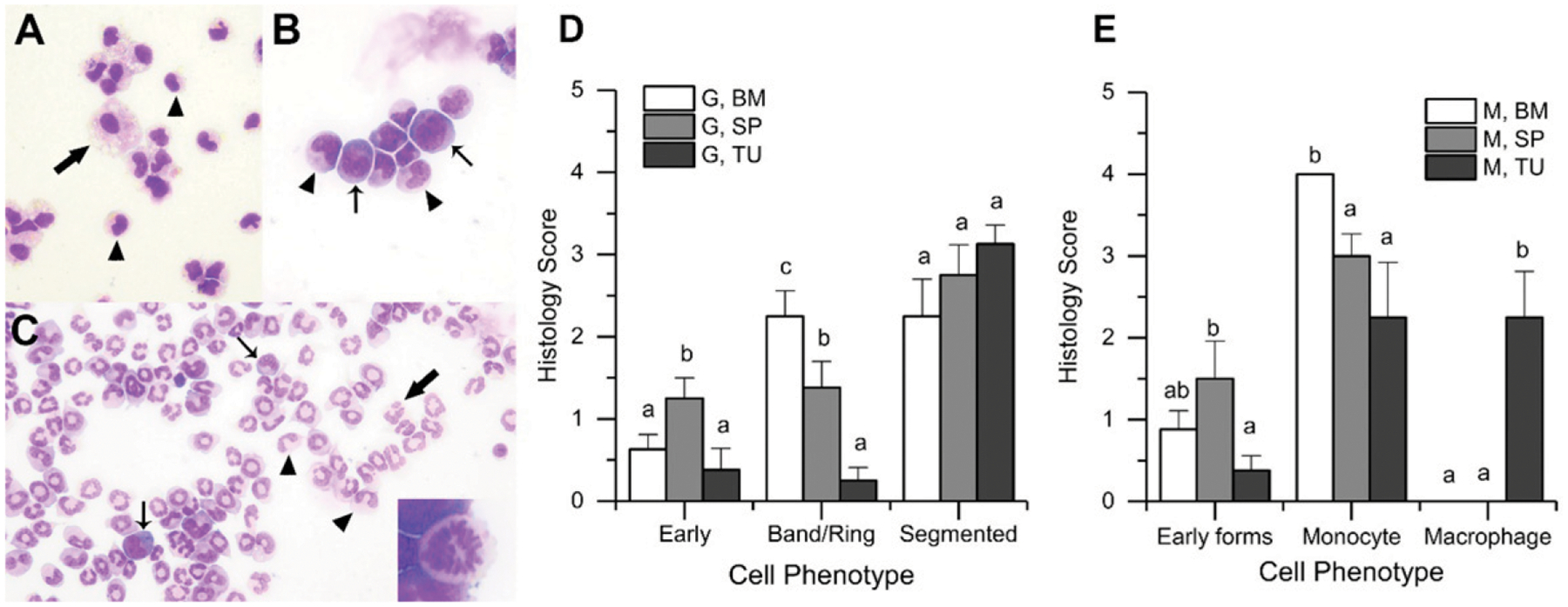
Morphologic and functional differences among MDSCs from bone marrow, spleen, and tumors (**A**) Representative images of tumor M-MDSCs showing cells with monocytic features (arrowheads) of oval, bilobed, or reniform nuclei and variably vacuolated cytoplasm as well as cells with macrophage features (arrow) of larger cells with abundant cytoplasm and extensive cytoplasmic vacuolation (Modified Wright-Giemsa, original magnification ×1000). (**B**) Spleen M-MDSCs showing cells with monocyte features (arrowheads) and cells with immature myeloid features of bluer cytoplasm and a higher nucleus-to-cytoplasm ratio (thin arrows) (Modified Wright-Giemsa, original magnification ×1000). (**C**) Tumor PMN-MDSCs showing cells with features of segmented neutrophils (thick arrow), band neutrophils (arrowheads), and more immature forms such as myeloblasts and metamyelocytes (thin arrows). The insert depicts a mitotic figure found elsewhere on the slide (Modified Wright-Giemsa, original magnification ×400). (**D** and **E**) Summaries of the distribution of cellular phenotypes in PMN-MDSCs (= G) (D) or M-MDSCs (E) from bone marrow (BM), spleen (SP), and tumor (TU) of mice with 4T1 tumors. Bars represent the mean ± SEM of scores from *n* = 8 mice. Different lowercase letters within a phenotype category are significantly different from one another (*p* < 0.05, Kruskal–Wallis test).

Because MDSCs have been described as immature myeloid precursors (10, 33), we sought to determine whether there would be morphologic and developmental heterogeneity among MDSCs isolated from separate compartments. When cytospins were scored by a certified pathologist (G.N.B.) in a double-blinded fashion, significant differences were observed in the cellular phenotype of MDSCs isolated from different tissues (Fig. 1). We found that there were very few cells with precursor features in the tumor PMN-MDSC population (Fig. 1C, 1D; *p* < 0.001 versus BM and spleen for band/ring forms), whereas >50% of the spleen and BM PMN-MDSC populations were characterized by immature cell features (Fig. 1D). Similarly, tumor M-MDSCs had a large number of cells with features of mature macrophages (Fig. 1A, 1E; *p* < 0.001 versus BM and spleen), whereas spleen and BM had a significant presence of immature/early cell forms, early forms (e.g., blue cytoplasm, higher N:C ratio, mitotic figures), and monocytic features (Fig. 1B, 1E). Monocytic forms were highest in M-MDSCs from the BM (>75%) and lowest in tumor (<50%) (*p* = 0.0102) (Fig. 1A, 1E). Predominant detection of morphologically immature myeloid cells within BM and spleen was anticipated based on the current literature description of MDSCs as immature. However, the data demonstrating that myeloid cells from the tumor possess very mature morphology are incongruous with the current literature stating that tumor MDSCs should also be immature. These results led us to hypothesize that the immature cells from the BM and spleen should have the ability to differentiate into multiple myeloid cell types, whereas this ability should be restricted in the more mature cells from the tumor. Therefore, we performed experiments to assess the potential of the monocytic myeloid cells from these compartments to differentiate into osteoclasts, granulocytes, macrophages, and DCs.

### Peripheral M-MDSCs and tumor M-MDSCs have different fate plasticity

We investigated how peripheral M-MDSCs and tumor M-MDSCs respond to optimal cytokine conditions for differentiation into other myeloid cell types and whether these responses can be modified by treatments that are intended to model the TME (i.e., addition of TES during culture).

#### Tumor-derived M-MDSCs do not convert to PMN-MDSCs.

**FIGURE 2.**
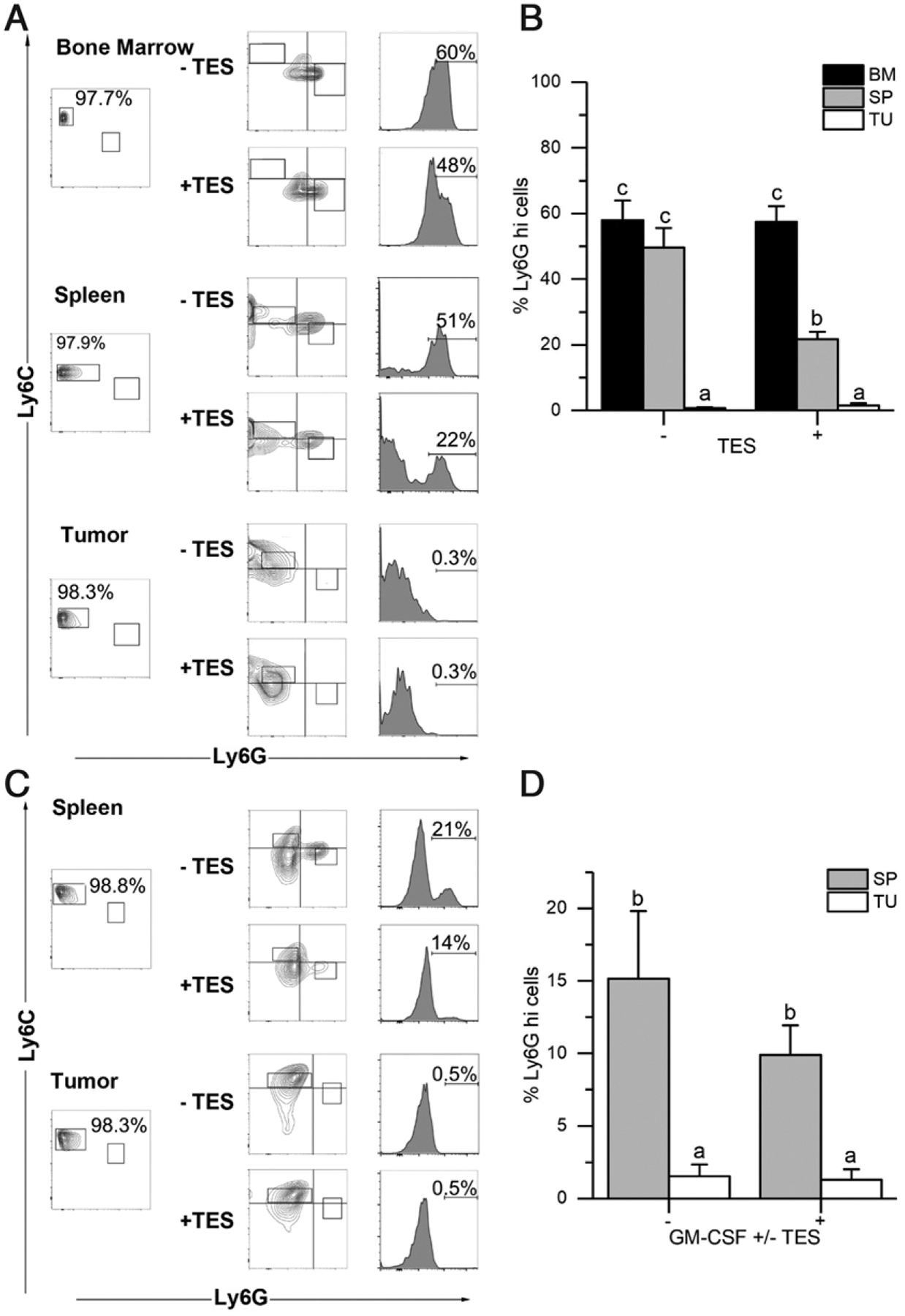
Tumor M-MDSCs do not differentiate into PMN-MDSCs M-MDSCs were isolated from bone marrow (BM), spleen (SP) and solid tumor (TU) of EL4 tumor-bearing mice. (**A–D**) Cells were cultured for 3 d with (A and B) basal medium (RPMI-C) or RPMI-C + tumor extract supernatant (TES), or (C and D) GM-CSF or GM-CSF + TES and then harvested from plates and stained for FACS analysis. (A and C) Representative biplots of postisolation purity of M-MDSCs from BM, SP, and TU (left), postculture Ly6G × Ly6C biplots (center), and histogram of the Ly6G signal distribution (right). (B and D) Summary graphs showing the percentage of M-MDSCs that gained Ly6G expression in the experiment. Bars represent the mean + SEM for (B) *n* = 5 or (D) *n* = 8 biological replicates for each tissue. Statistical analysis done on transformed data (natural log) using mixed design ANOVA. Values with different lowercase letters are significantly different from one another (*p* < 0.05, Tukey’s HSD test).

Others have shown that spleen or BM M-MDSCs can convert to a PMN-MDSC phenotype (loss of Ly6C, gain of Ly6G) when cultured for 3 or 5 d with GM-CSF and TES (14, 25). To examine whether this capability was also inherent in tumor M-MDSCs, we first asked whether the shift toward the PMN-MDSC phenotype was a general feature of M-MDSCs. In a basal medium (no GM-CSF or TES), BM and spleen M-MDSCs from mice bearing EL4 tumors had a strong shift toward the PMN-MDSC phenotype (58 and 50%, respectively) whereas tumor M-MDSCs did not gain Ly6G expression (<1%) (Fig. 2A). We obtained similar findings with M-MDSCs from two additional tumor models, 4T1 and RM1 (Supplemental Fig. 2). The presence of TES did not significantly alter the shift of BM M-MDSCs toward PMN-MDSCs (58%). However, TES significantly reduced the ability of spleen M-MDSCs to gain the PMN-MDSC phenotype (from 50 to 21.6%, Fig. 2B); this shift was still significantly greater than what we observed for tumor M-MDSCs (1–1.5% in the absence or presence of TES). In a separate series of experiments, we cultured spleen or tumor M-MDSCs for 3 d in GM-CSF with or without 20% TES, after which we harvested them for FACS analysis (Fig. 2C, 2D). Similar to what we observed for TES alone (Fig. 2B), spleen M-MDSCs treated with GM-CSF alone had a significant shift toward PMN-MDSC–like cells (15%) whereas tumor M-MDSCs did not respond to this stimulus (1.5%) (Fig. 2B). The combination of GM-CSF with TES caused a nonsignificant reduction in the shift of spleen M-MDSCs toward the PMN-MDSC phenotype (from 15 to 10%) whereas tumor M-MDSCs were still unresponsive (1.3%) (Fig. 2D).

#### Tumor M-MDSCs are resistant to becoming DCs.

**FIGURE 3.**
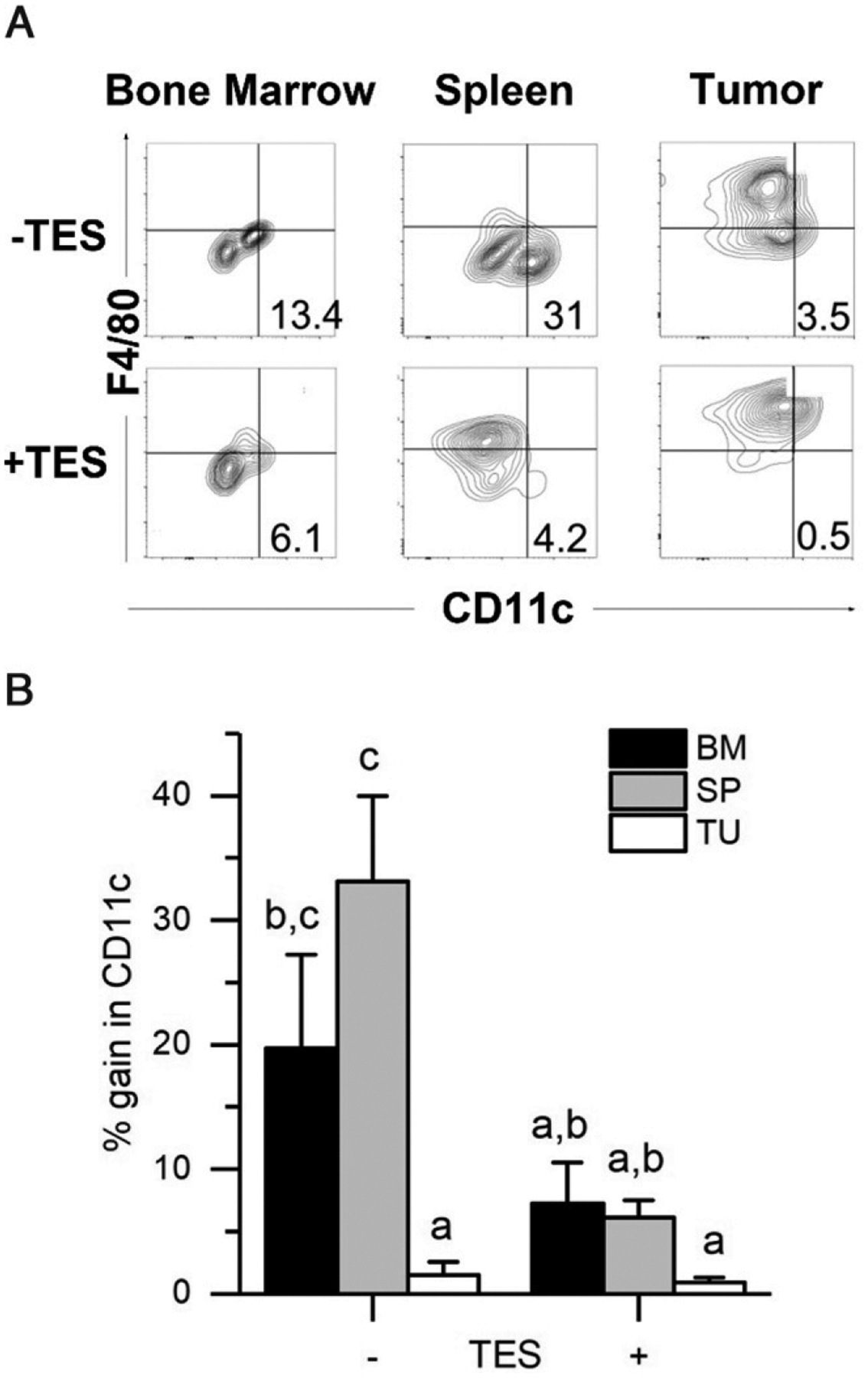
Tumor M-MDSCs do not respond to differentiation signals for dendritic cells M-MDSCs were isolated from bone marrow (BM), spleen (SP), and solid tumor (TU) of EL4 tumor-bearing mice and cultured for 3 d in the presence of GM-CSF and IL-4, with and without TES, and then analyzed by flow cytometry for markers of dendritic cells (CD11c). (**A**) Representative flow cytometry plots of post–GM-CSF and IL-4 culture. (**B**) Summary plot of CD11c^+^ cells. Bars represent the mean ± SEM for *n* = 3 biological replicates for each tissue. Statistical analysis done on transformed data (natural log) using mixed design ANOVA. In (B), values with different lowercase letters are significantly different from one another (*p* < 0.05, Tukey’s HSD test).

Several studies have suggested that MDSCs are a precursor for inflammatory DCs (34, 35), and a recent review suggested that M-MDSCs can differentiate into DCs at the tumor site (36). Therefore, we asked whether BM, spleen, and tumor-derived M-MDSCs could differentiate into DCs under standard conditions (GM-CSF + IL-4) and whether the presence of TES would alter the ability of M-MDSCs to become DCs (Fig. 3). GM-CSF 1 IL-4 treatment caused BM (19.7 ± 7.5% of cells) and spleen (33.1 ± 6.9% of cells) M-MDSCs to significantly gain CD11c expression whereas tumor M-MDSCs were unresponsive to these treatments (1.5 ± 1.0% of cells) (Fig. 3B). Similar effects were seen in BM and spleen M-MDSCs from the 4T1 and RM1 tumor models (Supplemental Fig. 3A, 3B). TES treatment significantly reduced the ability of BM (7%) and spleen (6%) M-MDSCs to gain CD11c compared with cytokine alone (Fig. 3B, *p* < 0.05) whereas TES-treated, tumor-derived M-MDSC remained unable to gain CD11c expression (<1%).

#### M-MDSCs from all three sites efficiently differentiate into F4/80^+^ macrophages.

**FIGURE 4.**
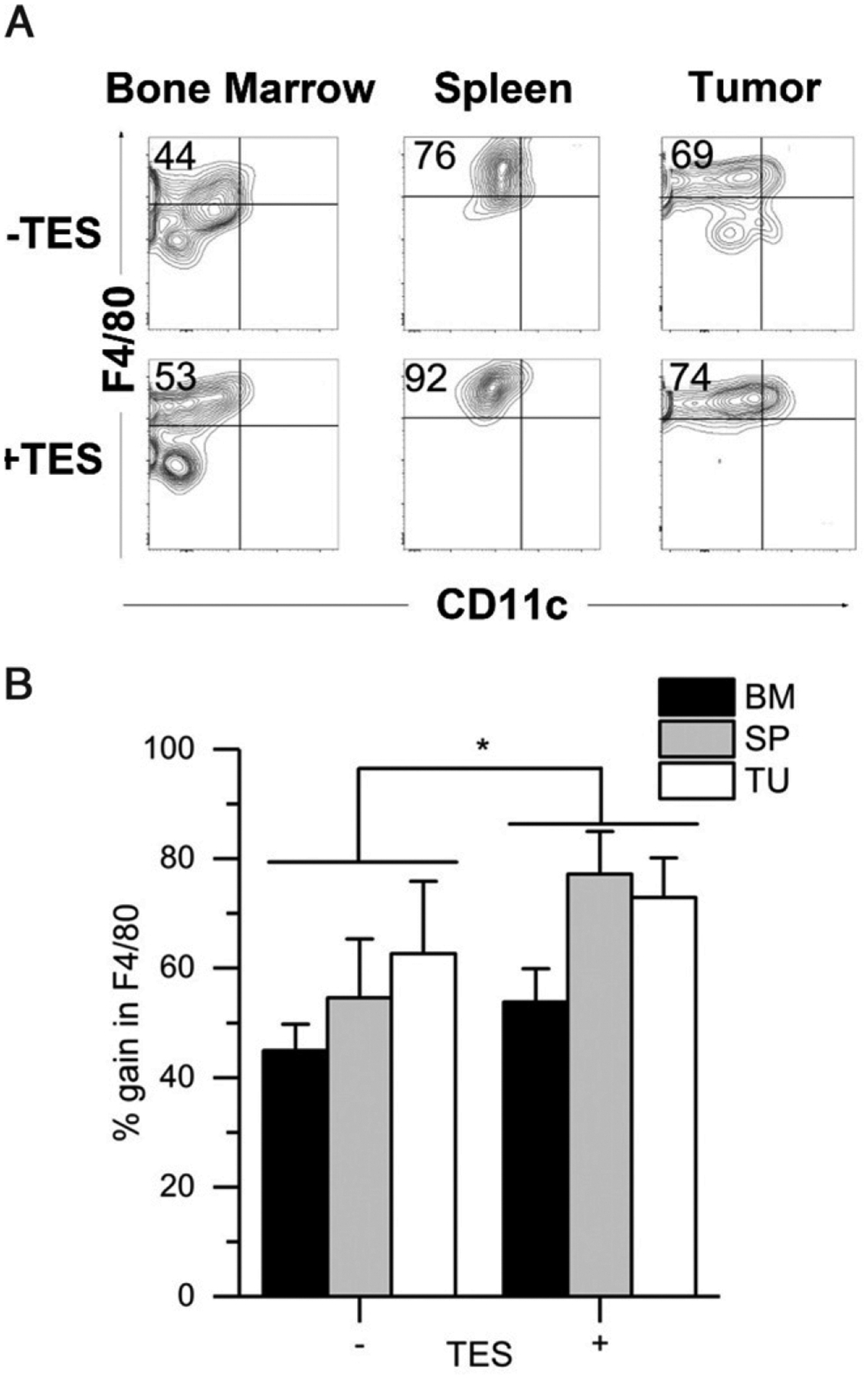
Tumor, spleen, and bone marrow M-MDSCs respond to differentiation signals for macrophages M-MDSCs were isolated from bone marrow (BM), spleen (SP), and solid tumor (TU) of EL4 tumor-bearing mice and cultured for 3 d in the presence of M-CSF with and without TES and then analyzed by flow cytometry for the macrophage marker F4/80. (**A**) Representative flow cytometry plots of post–M-CSF culture. (**B**) Summary plot of F4/80^+^ cells. Bars represent the mean ± SEM for *n* = 3 biological replicates for each tissue. Statistical analysis was performed on transformed data (natural log) using mixed design ANOVA. In (B), + (with) TES versus – (without) TES. *p < 0.05, Tukey’s HSD test.

In the TME, tumor-associated macrophages have been identified as F4/80^+^ cells, whereas M-MDSCs at the tumor site are F4/80-negative (Supplemental Fig. 1). Recently, Kumar et al. (6) reported that adoptively transferred BM M-MDSCs are more likely to differentiate into tumor-associated macrophages when they enter the tumor site (60%) than the spleen (20%). To compare the ability of BM, spleen, and tumor M-MDSCs to differentiate toward macrophages, we treated cells with M-CSF ± TES. In the presence of M-CSF alone, the M-MDSCs from all three tissues significantly gained F4/80 expression (Fig. 4), with tumor and spleen M-MDSCs having the highest ability with a 54.6 and 62.7% conversion rate, respectively, compared with BM (40% conversion), although these differences were not statistically different (Fig. 4B). A similar robust conversion of M-MDSCs from BM, spleen, and tumor to F4/80^+^ macrophages was seen using cells from the RM1 and 4T1 tumor models (Supplemental Fig. 3C–E). The addition of TES significantly increased the amount of M-MDSCs that converted to F4/80^+^ cells across all tissues (*p* = 0.0005, Fig. 4B).

#### Tumor-derived M-MDSCs are resistant to osteoclast differentiation.

**FIGURE 5.**
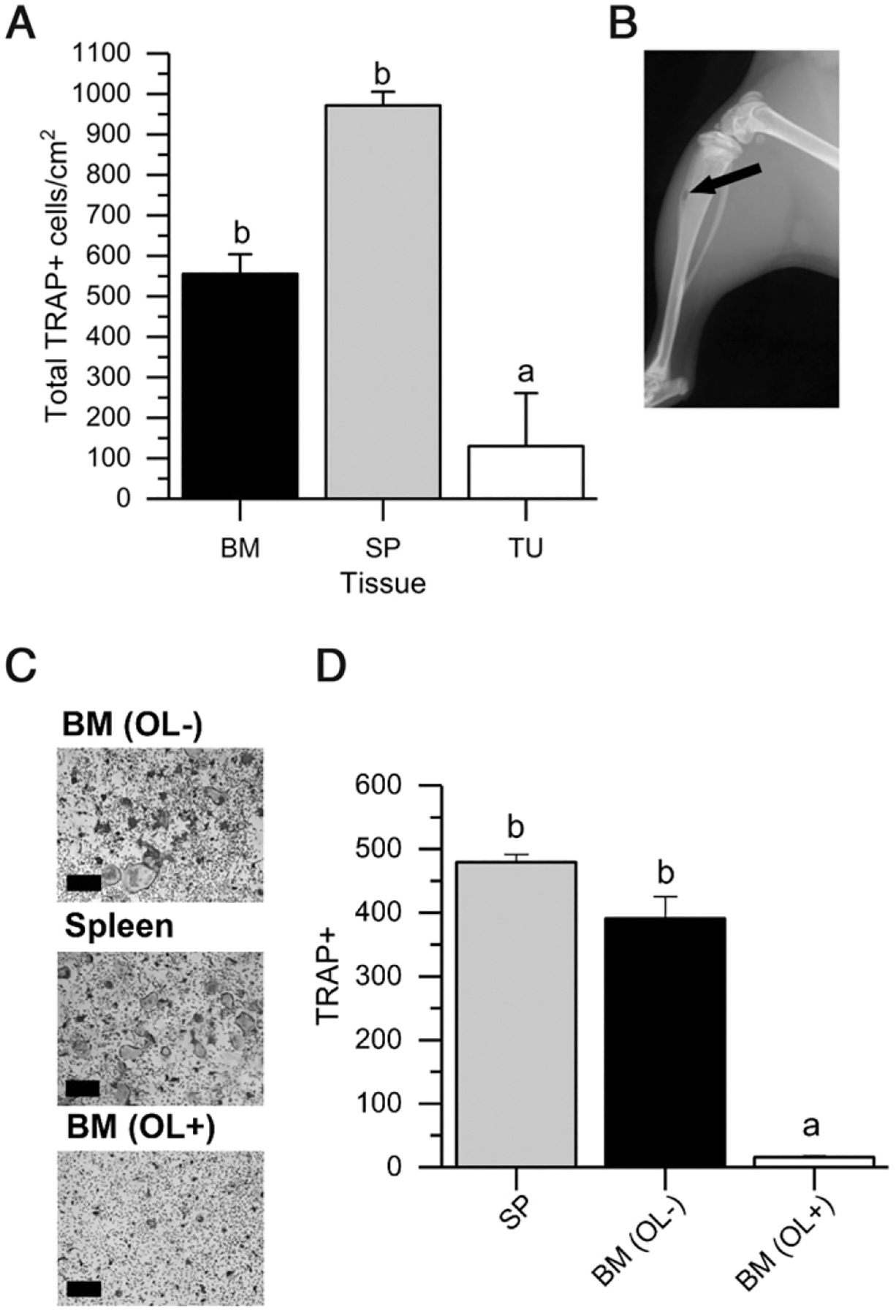
Tumor M-MDSCs do not respond to osteoclast differentiating signals (**A**) M-MDSCs from bone marrow (BM), spleen (SP), and solid tumor (TU) were isolated from 4T1 tumor-bearing mice and cultured 8 d with M-CSF and RANKL. TRAP^+^ cells were counted after culture. (**B–D**) Mice received an intracardial injection of 1 × 10^5^ 4T1 cells. After 10 d, femora and tibias were x-ray imaged. M-MDSCs were then collected by harvesting BM from bones showing osteolysis (OL+), contralateral bones with no osteolysis (OL−), or the spleen of the tumor-bearing mice. M-MDSCs were cultured for 8 d with M-CSF and RANKL and TRAP^+^ cells were counted. (B) Representative x-ray of osteolytic site on tibia. (C) Representative microscopy images (TRAP staining, original magnification ×200), showing large, TRAP^+^ osteoclasts in M-MDSC cultures from OL− BM, OL+ BM, and spleen. (D) Graph of TRAP^+^ cells following the treatment period. Bars represent the mean + SEM (*n* = 3). Values with different lowercase letters are significantly different (*p* < 0.05, Tukey’s multiple comparison test).

Treating total MDSCs from BM or spleen of tumor-bearing mice with M-CSF and RANKL induces their differentiation into TRAP^+^ osteoclasts (22, 37), but the ability of tumor M-MDSCs to become osteoclasts has not been reported. Therefore, we examined the impact of M-CSF and RANKL treatment on the production of osteoclasts from tissue and tumor M-MDSCs of mice with 4T1 tumors. We found that the ability of BM-derived M-MDSCs to differentiate into osteoclasts was 7-fold greater than for spleen M-MDSCs. In contrast, tumor M-MDSCs showed little ability to differentiate into osteoclasts (80% lower than spleen, Fig. 5A). Similar results were obtained using M-MDSCs from mice with RM1 and EL4 tumors (Supplemental Fig. 4).

### The TME reprograms differentiation potential

Owing to the stark differences in in vitro differentiation potential of peripheral versus tumor MDSCs, we hypothesized that the TME would reprogram peripheral MDSCs to have reduced osteoclastic potential. To test this hypothesis, we used a model of bone metastases caused by the intracardial injection of 4T1 breast cancer cells. This leads to the formation of tumors in the femora and tibia that cause OLs (Fig. 5B). M-MDSCs from three locations were examined for their ability to become osteoclasts: 1) the BM of femora with tumors and OLs (OL+), 2) the BM of contralateral femora lacking OLs (presumed to be tumor free [OL−]), and 3) the spleen. As we previously observed with tissue M-MDSCs from 4T1 tumor-bearing mice, there was a high degree of osteoclast formation from M-MDSCs isolated from spleen and from OL− BM (Fig. 5C, 5D). In contrast, M-MDSCs from OL+ BM that are closely associated with metastatic bone tumors developed very few small osteoclasts in response to the differentiation stimuli. These data indicate that the TME reprogrammed the BM M-MDSCs and made them resistant to osteoclast differentiation signals.

Importantly, our in vitro differentiation studies were consistent in two different mouse strains (BALB/c and C57BL/6), three different tumor models (4T1, RM1, EL4), and male or female mice. Additionally, the in vivo osteoclast studies demonstrating inhibition of M-MDSC differentiation in the presence of tumors are consistent with our in vitro observations and add substantial rigor to our results. Furthermore, these differentiation studies are consistent with the cytology studies in demonstrating that whereas BM and splenic M-MDSCs appear and behave as immature cells, the tumor MDSCs seem to be a mature cell. Because of the clearly defined differentiation potentials between peripheral MDSCs and those from the tumor, we hypothesized that there would be unambiguous functional differences in their suppressive capacities immediately after isolation.

### Only tumor-derived M-MDSCs suppress T cell proliferation when assessed immediately upon isolation

**FIGURE 6.**
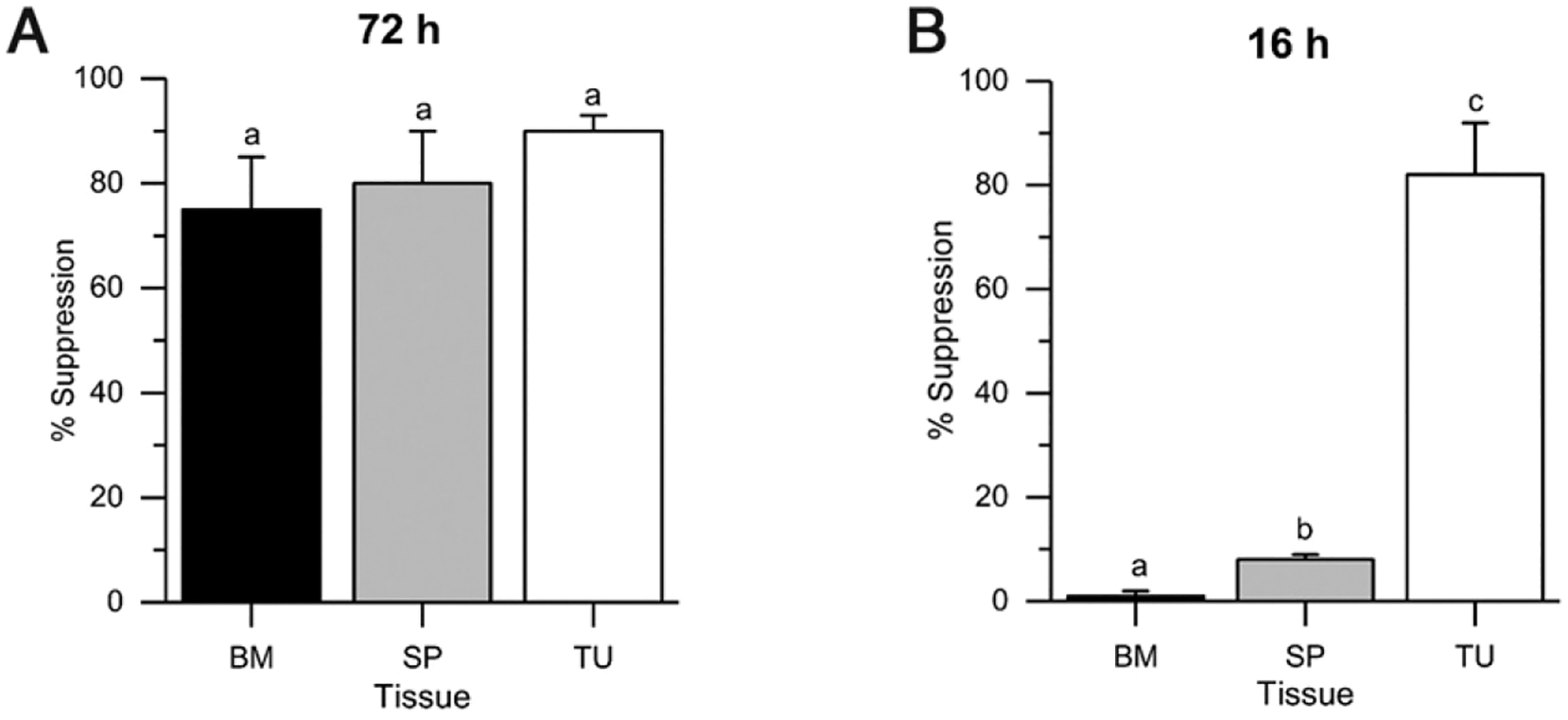
Splenic M-MDSCs from tumor-bearing mice acquire suppressive ability in vitro (**A** and **B**) M-MDSCs were isolated from bone marrow (BM), spleen (SP), and solid tumors (TU) of 4T1 tumor-bearing mice and then cocultured with purified CD8^+^ T cells at a ratio of 1:1 for 72 h (A) or with preactivated CD8^+^ T cells for 16 h (B). Bars represent the mean + SEM for *n* = 3 observations per group. Bars with different lowercase letters are significantly different from one another (Tukey’s HSD test, *p* < 0.05).

Although many in the field of MDSC biology use a ≥48-h T cell suppression assay to show that M-MDSCs from BM, spleen, and tumor are all functional (11, 17, 38–41), we previously showed that unfractionated tumor CD11b^+^Gr-1^+^ MDSCs could suppress T cell proliferation immediately upon isolation in a short-term assay but that spleen MDSCs could not (12, 13). We now extend our previous studies to assess isolated M-MDSCs from various tissues for their immediate T cell suppressive ability. As expected from studies that used the gold standard 3-d suppression assay (10, 11, 38), we found that M-MDSCs from BM, spleen, and tumor of tumor-bearing mice can all suppress T cell proliferation in a 72-h suppression assay (Fig. 6A). However, when tested in a 16-h assay against preactivated T cells, only tumor M-MDSCs suppressed T cell proliferation whereas MDSCs isolated from spleen and BM were not suppressive (Fig. 6B). These data strongly suggest that peripheral tissue M-MDSCs do not have in vivo suppressive function. Our cumulative data demonstrate distinct differences between tumor M-MDSCs and those from peripheral sites to such an extent that we hypothesized that transcriptomic analysis would reveal substantial differences between cells from these two locations.

### The transcriptome of spleen MDSCs is dramatically different from tumor MDSCs

**FIGURE 7.**
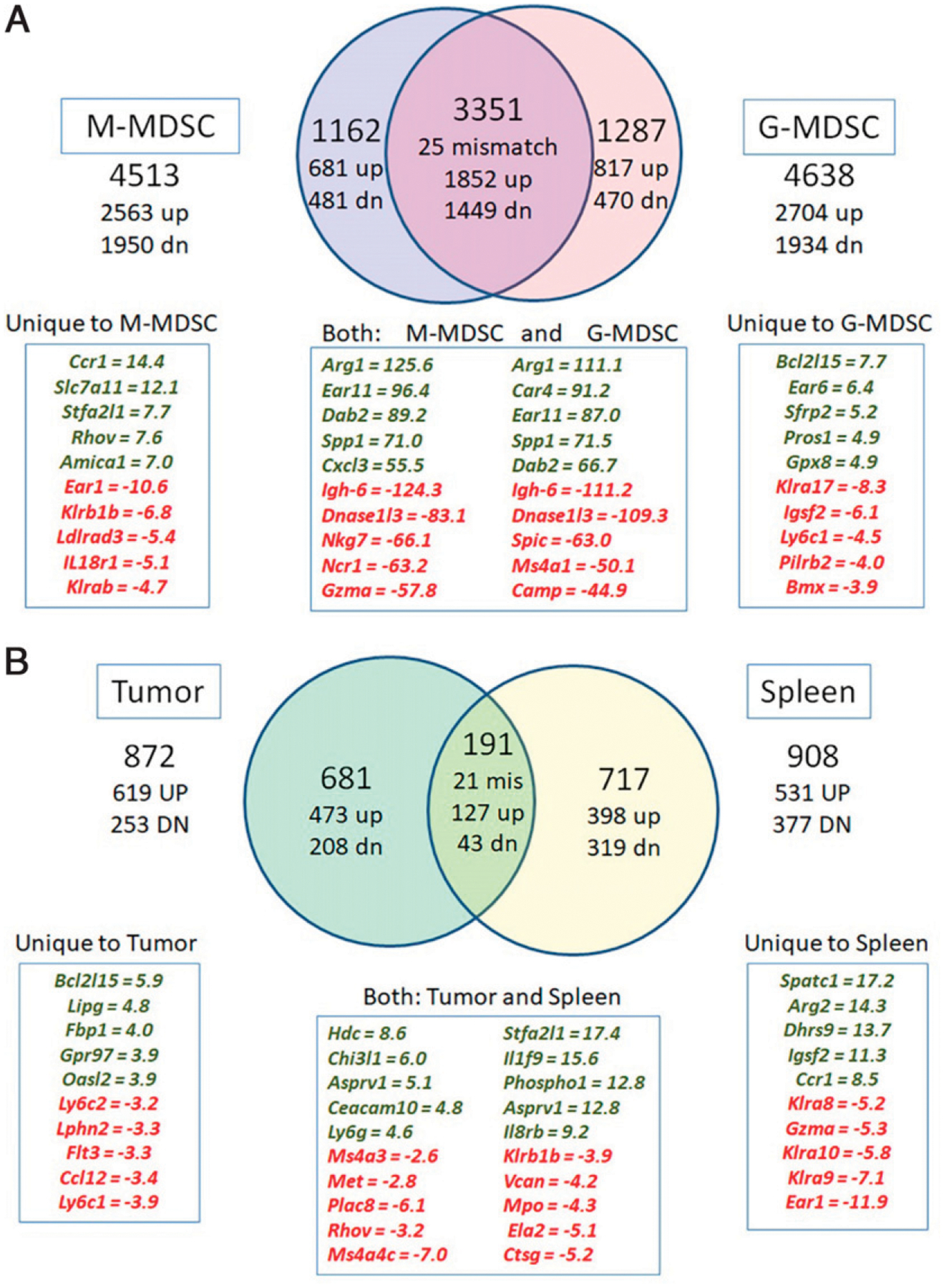
Spleen and tumor-derived MDSC subtypes have different gene expression patterns M-MDSCs and PMN-MDSCs (G-MDSCs) were isolated from the peritoneal ascites of mice with i.p. RM1 tumors or from the spleens of POET-3 mice with OT-1 cell–induced prostatic inflammation. RNA was isolated, and the transcript profile of each spleen or tumor MDSC subtype was assessed by Affymetrix microarrays. (**A**) Venn diagram of the differentially expressed genes (DEGs, 1.5 FC, 5% FDR) identified as M-MDSCs (left) or PMN-MDSCs (right) that transitioned from spleen to tumor MDSCs (*n* = 4 biological replicates per group). A list of the top five upregulated or downregulated DEGs for spleen-to-tumor transition for both M-MDSCs and PMN-MDSCs (center) or for each subtype is shown. (**B**) Venn diagram of the DEGs (1.5 FC, 5% FDR) identified between M-MDSCs and PMN-MDSCs within the spleen (right) or tumor (left) (*n* = 4 biological replicates per group). A list of the top five upregulated or downregulated DEGs for the M-MDSC to PMN-MDSC comparison (center) or for each tissue type is shown.

We previously reported that MDSCs isolated from the tumor or inflamed prostates have high expression levels of Arg1 and Nos2 mRNA but that neither CD11b^+^Gr-1^+^ MDSCs isolated from the periphery of tumor-bearing mice nor MDSC subtypes (M-MDSc, PMN-MDSCs) isolated from the periphery of mice with inflamed prostates express these genes (12). We further explored the molecular differences that define spleen and tumor MDSC subtypes using microarray analysis. Of the 22,392 probe sets analyzed, 6,228 probe sets representing 5,550 unique gene names were differentially expressed in at least one of the four pairwise comparisons we conducted (fold change [FC] > 1.5-fold at 5% FDR). A large number of genes were differentially expressed during the transition of PMN-MDSCs (*n* = 4638) or M-MDSCs (*n* = 4513) from the spleen to the tumor environment (Fig. 7A). Seventy-five percent of the genes that were differentially expressed between spleen and tumor MDSCs were common to both subtypes (Fig. 7A), including the classical tumor MDSC markers Arg1 (>100-fold increased) and Nos2 (16- to 32-fold increased). In addition to Arg1 (the top upregulated genes for both G-MDSCs and M-MDSCs), the top upregulated genes common to both subtypes are shown in Fig. 7A. There were also transcripts that uniquely defined the transition from spleen to tumor for M-MDSCs (*n* = 1162) or PMN-MDSCs (*n* = 1287); the top subtype-specific genes are shown in Fig. 7A. Similarly, there are a number of genes that are differentially expressed between PMN-MDSCs and M-MDSCs in the tumor environment (*n* = 872, Fig. 7B). The level of differential gene expression between tumor G-MDSC and M-MDSC subtypes is lower than the level seen when the MDSC subtypes transition from spleen to tumor (e.g., the highest upregulated gene is Hdc at 8.6-fold higher in G-MDSCs versus M-MDSCs). These differentially expressed tumor transcripts are distinct from those that define the difference between the MDSC subtypes in the spleen (*n* = 908 with only 170 that overlap with the tumor subset comparison).

To gain insight into the functional changes that characterize the development of spleen MDSCs into tumor MDSCs, as well as the difference in G-MDSC and M-MDSC subtypes in the tumor environment, we conducted bioinformatics analysis for the comparisons reflected in Fig. 7A. A summary of these analyses is reported in Table I. The most prominent enriched pathway that was common to the spleen-to-tumor transition for both MDSC subtypes was for HIF-1 signaling and this included upregulation of HIF-1 mRNA by 3- (PMN-MDSCs) to 3.9-fold (M-MDSCs). Similarly, upregulation of an expression network centered on CREB1 and downregulation of a network centered on NF-κB were prominent in the differentially expressed genes common to both subtypes during the spleen-to-tumor transition. Signatures specific to the transition into a tumor M-MDSC included the following: downregulation of cell cycle, upregulation of chemotaxis/Ccl2 signaling, and activation of pathways for myeloid and macrophage differentiation. Signatures more specific to the transition into a tumor PMN-MDSC include the following: upregulation of pathways for cell matrix interactions or extracellular matrix remodeling, upregulation of ATP/inosine 5′-triphosphate metabolism, and upregulation of a network centered on p53. From these data combined with those presented above, we conclude that splenic MDSCs are a very different cell versus tumor MDSCs, which generates significant concern for using them as surrogates for studying myeloid cell suppression in the TME. However, we wondered whether using a common method of mimicking the TME in vitro, that is, adding TES to spleen M-MDSC cultures, might cause the splenic M-MDSCs to transition to a tumor M-MDSC character.

### TES induces only a partial conversion of spleen M-MDSCs to tumor M-MDSCs

A popular in vitro method used to model MDSCs is culturing BM or spleen MDSCs in tumor-mimicking conditions modeled by the addition of TES. However, it is not known whether TES treatments can transform peripheral MDSCs into tumor MDSC-like cells.

**FIGURE 8.**
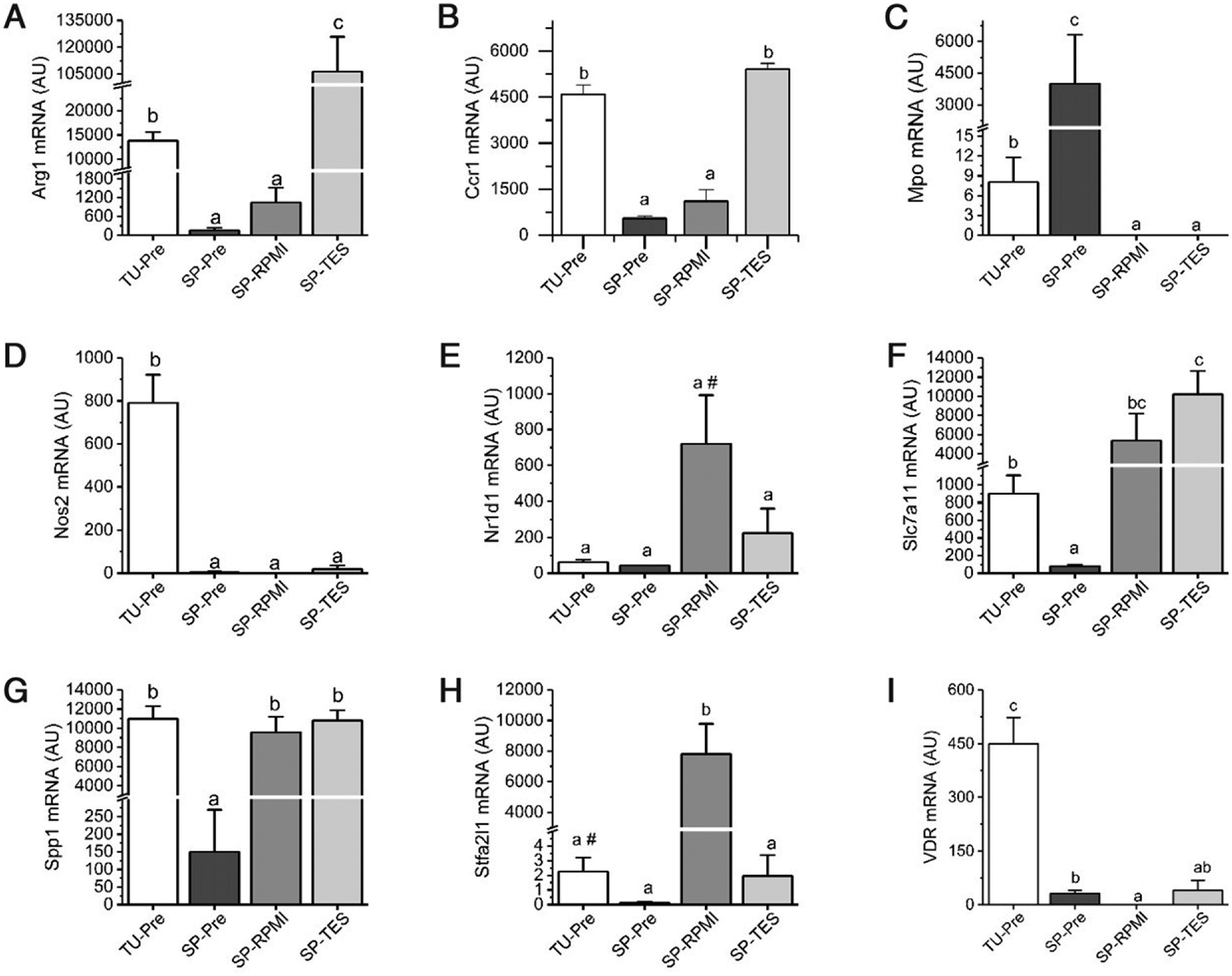
Expression of tumor M-MDSC marker genes in in vitro–generated M-MDSCs is inconsistent with full conversion to tumor M-MDSCs (**A–I**) M-MDSCs were isolated from tumors (TU-Pre) and from spleens (SP-Pre) of EL4 tumor-bearing mice and RNA from these cells were analyzed as a reflection of the cell state within the mouse at the time of harvest (i.e., preculture). Spleen M-MDSCs were cultured for 48 h in control medium (SP-RPMI) or medium containing EL4 tumor extract supernatant (SP-TES). RNA was analyzed for mRNAs found to be differentially expressed between spleen and tumor M-MDSCs in our microarray experiment. Bars represent the mean ± SEM (*n* = 3–5 biological replicates per sample). Within each panel, bars with different lowercase letters were significantly different from one another (*p* < 0.05, Fisher’s protected least significant difference test). ^#^*p* < 0.1 versus SP-Pre.

To test the validity of the in vitro TES model, we examined the impact of TES treatment (72 h) in cultured spleen M-MDSCs on the expression of nine genes that our array analysis revealed to be differentially expressed during the transition of spleen M-MDSCs to tumor M-MDSCs (Fig. 8). The differentiation-induced change in expression of eight of these genes was confirmed (i.e., comparison of freshly isolated M-MDSCs from spleen versus tumor); only the downregulation of Nr1d1 mRNA in tumor M-MDSCs was different from what we observed in the microarray experiment. In vitro culture of spleen M-MDSCs in RPMI-C alone had variable effects on the expression of the nine genes. As crucial genes associated with suppression, Arg1, Ccr1, Nos2, and Vdr mRNA levels did not change in cultured spleen M-MDSCs whereas Mpo mRNA levels were reduced and Slc7a11, Spp1, and Stfa2l1 mRNA levels were elevated. Treatment of spleen M-MDSCs with RPMI-C + TES induced the expression of Arg1 (to 10-fold greater than tumor M-MDSCs) and Ccr1 mRNA but had no effect on the expression of Mpo, Nos2, Slc7a11, Spp1, or Vdr mRNA and reduced the expression of Stfa2l1 mRNA levels. These data, combined with data on the impact of TES treatment on differentiation shown in Figs. 2–4, demonstrate that the impact of TES treatment on spleen or BM M-MDSC differentiation is limited and incomplete and did not induce Nos2 expression, an important gene linked to the suppressive activity of freshly isolated M-MDSCs (18).

## DISCUSSION

The current, accepted model of MDSCs defines the cells from spleen of tumor-bearing hosts and from tumor as nearly equivalent (9, 36). However, our results demonstrate that significant phenotypic and functional differences exist between cells from the two compartments, including differences in their morphology, transcript profile, fate plasticity, and T cell suppressive ability. These data are consistent with the hypothesis that tumor M-MDSCs are a pathologic cell rather than an immature cell that has been activated in a pathological environment. This hypothesis is supported by studies that demonstrate functional differences in T cell suppressive capacity between spleen- and tumor-derived MDSCs (6, 11, 42). Furthermore, the data reported in the present study, in conjunction with previous reports showing differences in suppressive activity between spleen and tumor M-MDSCs, suggest that studies using M-MDSCs isolated from nontumor, peripheral sites do not accurately reflect tumor-resident M-MDSC biology. We contend that to understand the role of M-MDSCs in cancer, they must be studied from the pathological site where they are functionally active, that is, the TME.

The function of MDSCs is defined by their ability to suppress T cell proliferation or IFN-γ production (9). This suppression is assessed via assays that are typically longer than 48 h (11, 17, 38–41, 43–45). However, our data show that the results from long-term T cell suppression assays do not expose an important aspect of M-MDSC suppressive function. By using a short-term assay (12–16 h) with preactivated T cells that better reflects the intrinsic in vivo function of the isolated MDSCs, our data show that only tumor-derived MDSCs can suppress T cell proliferation and that MDSCs from peripheral sites are not functional upon isolation and by inference are not functional in vivo (12) (13) (Fig. 6B). The acquisition of suppressive function in the longer 48- to 72-h assay is at least partly due to the activation of BM and spleen cells by the IFN-γ released by activated T cells (12, 46). Therefore, we propose using the 16-h short-term assay as the gold standard for testing and identifying the T cell suppressive function of candidate MDSC populations.

Our microarray analysis reveals large differences in the transcriptome of MDSCs from spleen and tumor. This observation is consistent with two published studies. One compared the transcript profile of CD11b^+^Ly6G^+^ cells from spleen and tumor of BALB/cJ mice with AB12 tumors (47). We reanalyzed these data and found 5642 unique, differentially expressed genes (10% FDR); 2059 of these transcripts overlapped with our list of differentially expressed genes for the transition of PMN-MDSCs from the spleen to the tumor. The overlap includes most of the highly, differentially expressed genes we observed in both G-MDSCs and M-MDSCs (Fig. 7A), for example, Ccl12, Ccl17, Ear6, Pros1, Gpx8, Klra17, Ly6c1, Bmx, Arg1, Car4, Dab2, Spic, Camp, and MPO. A second publication compared the transcriptome of M-MDSCs (CD11b^+^Ly6C^++^Ly6G^−^) from influenza A–infected lung or spleen of infected mice (48). Of the 11,034 transcripts they detected in their sample, 2,346 were differentially expressed, including genes such as Ccl2, Il6, Tgfb3, and Nos2 from our list (Table I). Thus, our data, similar to those of the two previous studies, demonstrate that tumor MDSCs are not simply spleen MDSCs that have entered the tumor (or site of inflammation) but that they are cells that have undergone a vast molecular reprogramming consistent with differentiation. This interpretation is supported by our cytologic data demonstrating the appearance of mature myeloid cell phenotypes and the loss of early myeloid phenotypes in the tumor MDSC population compared with peripheral MDSCs. Furthermore, while not specifically identifying MDSCs, Zilionis et al. (19) convincingly demonstrated that circulating human myeloid populations have a transcriptomic profile that is distinct from the profile of tumor myeloid populations. We have extended the Zilionis et al. study by directly examining the molecular and functional differences of M-MDSCs from different mouse tissue environments. Our studies show such extensive differences between peripheral and tumor M-MDSCs that we conclude the disparities cannot be attributed to regulatory adjustments by an immature cell, but to reprogramming of peripheral M-MDSCs so that they differentiate to a more mature cell state.

A recent review by Condamine et al. (49) identified many potential transcriptional regulators of MDSC expansion and activation including IRF8, STAT1, STAT3, STAT6, C/EBPα, NF-κB, and Notch. Transcription factor networks identified in our data support a role for these transcriptions factors, as well as HIF1α (11), in the spleen-to-tumor transition. Our data also suggest that transcription factors such as CREB1 and ESR1 as well as pioneer factors such as PU.1 and Sp1 may contribute to the differentiation of MDSCs in the tumor. However, these findings require additional examination, especially as they relate to the differentiation of MDSC subtypes.

Cellular plasticity is another aspect of MDSC biology that also reveals important functional differences among M-MDSCs from BM, spleen, or tumor. The ability of BM or spleen M-MDSCs to differentiate toward mature myeloid cell phenotypes has been demonstrated in several in vitro studies (14, 20, 25, 50). However, it is difficult to compare these studies, and therefore assess the similarity of cells from different sites, because there is a lack of uniformity in the conditions used to induce differentiation. For example, in studies that examined the ability of spleen M-MDSCs to become PMN-MDSCs, macrophages, or DCs, some groups cultured in media alone (50, 51) whereas others culture cells in the presence of cytokines and/or tumor cell–conditioned media (11, 14, 25). More importantly, none of the studies on MDSC plasticity examined tumor M-MDSCs. As such, our study is novel, it directly compares M-MDSCs isolated from BM, spleen, and tumor under identical conditions and assess their ability to differentiate into other myeloid cell types. By doing this, our data reveal that tumor M-MDSCs have a very limited ability to become DCs, osteoclasts, or PMN-MDSCs compared with BM and spleen M-MDSCs (Figs. 2–5).

Our work on M-MDSCs and osteoclast differentiation, to our knowledge, is particularly intriguing. Metastatic tumor cells can migrate to bone and produce signals that promote osteoclast differentiation and cause bone loss (52). Previously, others have shown that tumors can reprogram BM or spleen MDSCs to become osteoclasts that contribute to tumor-induced bone osteolysis (20, 22). Our data extend these previous observations by showing that isolated tumor M-MDSCs have lost their ability to differentiate into osteoclasts, suggesting that metastatic bone tumors promote differentiation of BM M-MDSCs into the mature tumor M-MDSCs. Consistent with this hypothesis, we found that BM M-MDSCs lose their ability to differentiate into osteoclasts when they are in close proximity to experimentally induced bone tumors but not when they are in the contralateral bone lacking osteolysis. Thus, the role that M-MDSCs play in cancer-induced bone loss is complex and conditional upon the state of differentiation of the M-MDSCs.

Interpretations of previous studies contend that the spleen supplies the tumor with immunosuppressive protumor myeloid cells (53, 54), that is, splenic myeloid cells are precursors for MDSCs found in the tumor. Thus, although we find that freshly isolated spleen M-MDSCs are not suppressive in vivo, it is possible that they can acquire true M-MDSC functional character in vitro, for example, during the standard 72-h suppression assay or after treatment with TES, thereby making them useful as a model for M-MDSCs in the TME. However, several pieces of data argue against this interpretation and suggest that the in vitro transition of spleen M-MDSCs to tumor M-MDSCs is incomplete. First, spleen M-MDSCs studied in 72-h assays have lower T cell suppressive function than do tumor M-MDSCs in vitro and in vivo (11). This suggests that the signals from T cells in the suppression assay are not sufficient to drive complete differentiation of spleen M-MDSCs to tumor M-MDSCs, otherwise, the nature of suppressive function between the two sources would be similar. Second, our data demonstrate that signals from the tumor modeled by TES reduce the plasticity of BM and spleen M-MDSCs. However, although TES-treated BM and spleen M-MDSCs take on some tumor M-MDSC character, they are not identical to tumor M-MDSCs. For example, TES did not suppress the M-MDSCs to PMN-MDSC conversion of BM down to the low level of conversion that we observed for tumor M-MDSCs (Fig. 2D). Similarly, treatment of spleen M-MDSCs with TES induced only a subset of genes associated with freshly isolated tumor M-MDSCs (Fig. 7). Others have used multiple in vitro models to mimic the tumor environment and create cells with a tumor MDSC phenotype, including the use of tumor cell–conditioned media (55), TES (25), or purified cytokines (46), but researchers have not compared how M-MDSCs from different tissues respond to these conditions and few have directly compared in vitro–differentiated MDSCs to tumor MDSCs (56). In contrast, our data show that in vitro–differentiated M-MDSCs are not equivalent to tumor MDSCs and, as a result, we suggest caution when using these cells as a surrogate for tumor M-MDSCs. Importantly, however, note that our studies did not test two additional stimuli that have been demonstrated to be important for the development of functional tumor M-MDSCs. We did not model interactions between M-MDSCs and tumor cells (57), nor did we test for spleen M-MDSC differentiation in the hypoxic tumor environment (11). We have published that hypoxia does not induce splenic M-MDSCs to produce the NO required for suppression of T cell function nor did hypoxia induce splenic F4/80^+^ cells from tumor-bearing mice to gain T cell suppressive function in 18-h assays (18). Nonetheless, although we believe that our in vitro spleen M-MDSC studies accurately reflect the limits of the in vitro–differentiated spleen M-MDSC model, additional studies that directly test the impact of hypoxia and tumor–M-MDSC interactions are necessary to definitively test this position.

In summary, our data conflict with the model presented in recent reviews (36, 58) by providing evidence that cells with M-MDSC surface markers that are isolated from BM, spleen, and tumor represent different stages of M-MDSC development. This conflict is likely due to the fact that the model has been built on research that uses MDSCs isolated from spleen and BM. By carefully comparing MDSCs from peripheral and tumor sites, our data indicate that only tumor M-MDSCs are the true, functional M-MDSCs and that they are a mature cell with limited alternative cell fates. Taken together, our studies highlight the critical differences between peripheral and tumor M-MDSCs and may lead to the development of antitumor therapies and treatments with application to the clinic.

## Supplementary Material

Supplement

## Figures and Tables

**TABLE I.** Summary of functional enrichment analysis of MDSC array data

Group	Pathways	Network Hubs	Process Networks
Common to SP to TU transition across M-MDSC and PMN-MDSC subtypes			
Up	Transcription_HIF-1 targets	Sp1	Apoptosis: apoptotic mitochondria
	Oncostatin M signaling via Jak-Stat	CREB1	Chemotaxis
	PGE_2_ pathways in cancer	c-Myc	Proteolysis: ubiquitin proteasomal proteolysis
	Transport: clathrin-coated vesicle cycle	Jak/Stat5	Protein folding in normal condition/ER and cytoplasm
	IL-1 signaling pathway		
Down	IFN-α/β signaling via JAK/STAT	NF-κB	Cell cycle G_2_-M
	Ag presentation by MHC class I	Stat3	Ag presentation
			TCR signaling
Specific to M-MDSCs, SP to TU transition			
Up	ETV3 effect on CSF-1 promoted macrophage differentiation	CREB1	Chemotaxis
	PGE_2_ pathways in cancer		GO terms for localization and migration
	M-CSF-receptor signaling pathway		
Down	DNA damage ATM/ATR regulation of G_2_-M checkpoint	NF-κB	Cell cycle core/G_2_-M/S phase/mitosis
	Cell cycle: initiation of mitosis	Ubiquitin	
		CDK1	
Specific to PMN-MDSC, SP to TU Transition			
Up	Transcription: HIF-1 targets	CREB1	Integrin-mediated cell-matrix interactions
	Cell adhesion: ECM remodeling		Proteolysis: ubiquitin proteasomal proteolysis
	ATP/ITP metabolism		Regulation of EMT
	IL-1 signaling pathway		
	Clathrin-mediated cell adhesion		
Down	T cell cosignaling receptors	NF-κB	Phagocytosis
	IFN-α/β signaling via JAK/STAT	Jak/Stat	Phagosome in Ag presentation
		c-Myc	TCR signaling
TU PMN-MDSCs versus TU M-MDSCs			
G > M	Cell adhesion: ECM remodeling	p53	Cell cycle: G_2_-M
	Regulation of EMT	Tgfbr2	Proteolysis: ECM remodeling
	Cell cycle: initiation of mitosis		Connective tissue degradation
	Transcription regulation of granulocyte development		
M > G	G-CSF-induced myeloid differentiation	c-Myc	Innate inflammatory response
	CCL2 signaling/CCL2-induced chemotaxis	c-Src	Leukocyte chemotaxis

ATM, ataxia telangiectasia mutated; ATR, ataxia telangiectasia and Rad3-related; ECM, extracellular matrix; EMT, epithelial–mesenchymal transition; ER, endoplasmic reticulum; GO, Gene Ontology; ITP, inosine 5′-triphosphate; SP, spleen; TU, tumor.

## Abbreviations used in this article:

BM: bone marrow
DC: dendritic cell
DMEM-C: complete DMEM
FC: fold change
FDR: false discovery rate
G-MDSC: granulocyte-like MDSC
MDSC: myeloid-derived suppressor cell
M-MDSC: monocyte-like MDSC
OL: osteolytic lesion
PMN-MDSC: polymorphonuclear MDSC
POET-3: prostate OVA-expressing mice-3
RPMI-C: complete RPMI
TES: tumor explant supernatant
TM: tumor microenvironment
TRAP: tartrate-resistant acid phosphatase
