# Supplementary material for: Monocytic Myeloid-Derived Suppressor Cells from Tumor Tissue Are a Differentiated Cell with Limited Fate Plasticity
Source: Immunohorizons. Author manuscript; Available in PMC 2025 Mar 31. (PMC11955908; doi:10.4049/immunohorizons.2200079)
Supplement: Supplement [file NIHMS2068457-supplement-Supplement.pdf]

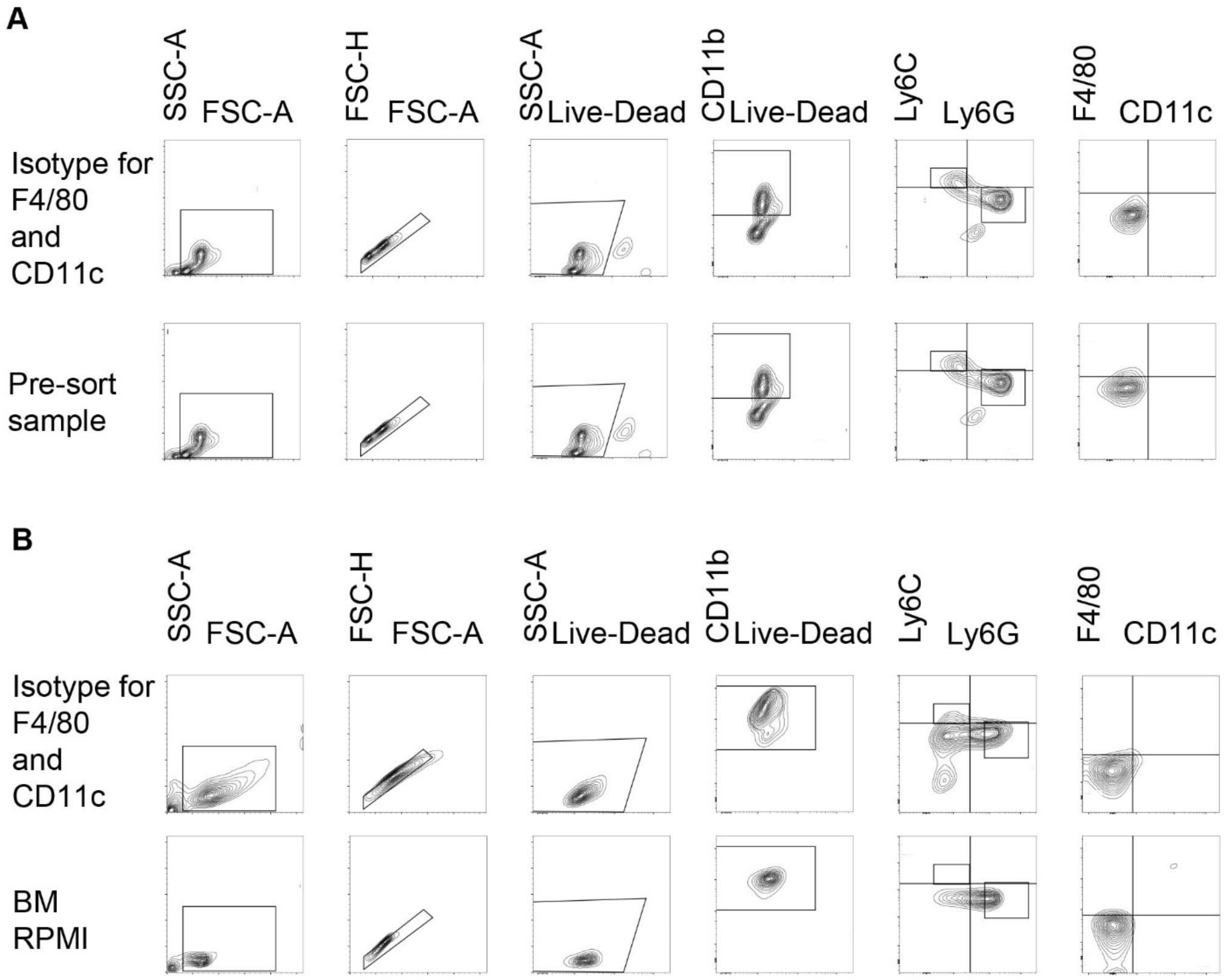

Figure S1: Gating strategies and sort purity for pre and post culture. Single cell suspension from bone marrow (BM), spleen (SP), and tumor (TU) of EL4 tumor-bearing mice were stained with antibodies for CD11b, Ly6C, Ly6G, F4/80 and CD11c. **(A)** Representative plots of gating strategy of pre-culture samples. Double isotype control staining methods were used to determine the F4/80 and CD11c expression prior to culture. **(B)** Representative plots of post culture gating strategy using the same method as pre-culture with isotypes to determine the F4/80$^-$, CD11c$^-$ gate.

Downloaded from http://journals.aai.org/immunohorizons/article-supplement/237579/pdf/immunohorizon_2200079_supplemental_1/ by guest on 24 March 2025

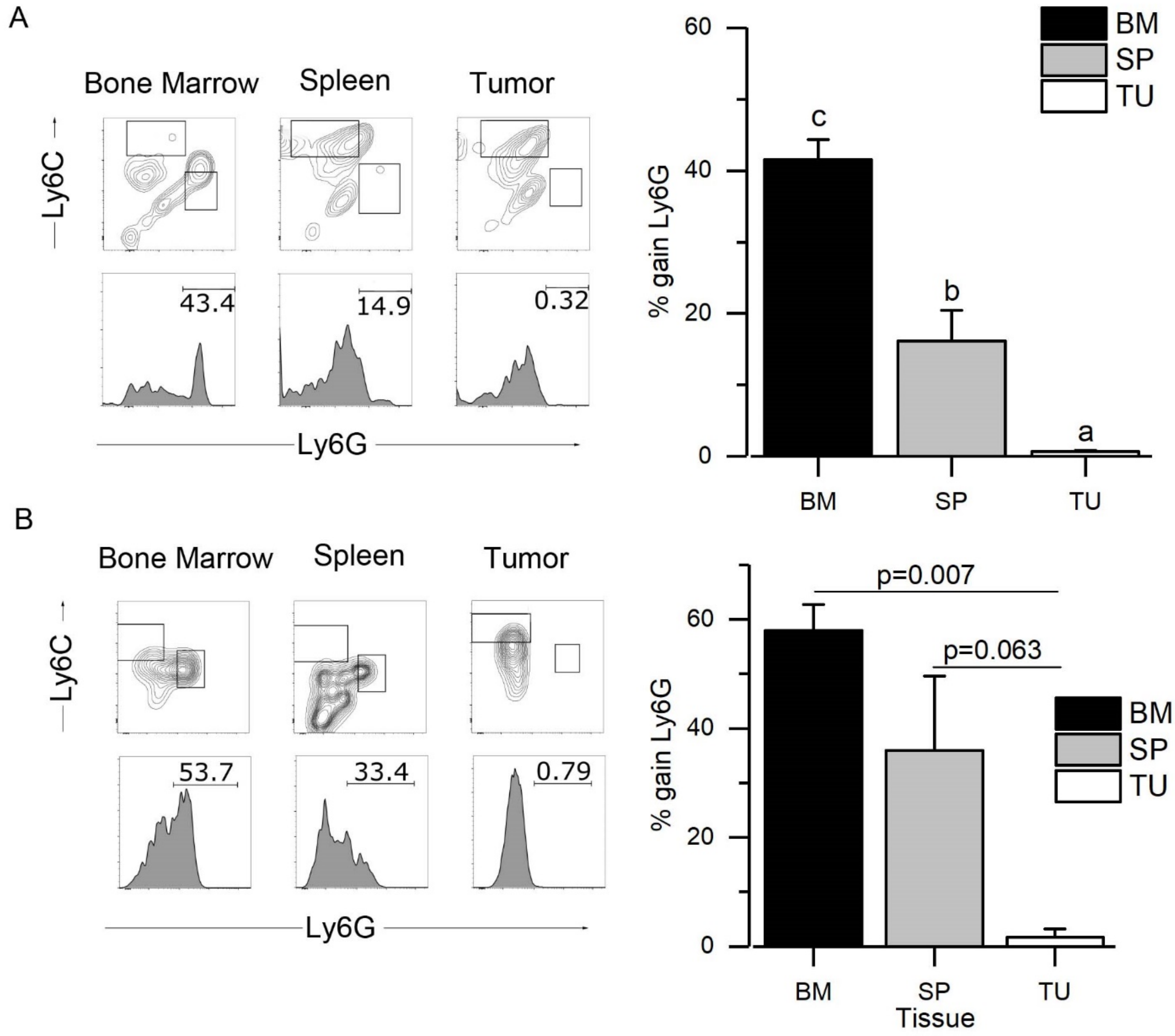

Figure S2: M-MDSC from RM1 and 4T1 tumor models confirm tumor M-MDSC do not become PMN-MDSC. 4T1 and RM1 tumors were allowed to grow for 14 d after sc injection then M- subtype cells were isolated from bone marrow (BM), spleen (SP) and solid tumor (TU) and cultured for 3 days in RPMI-C. After 3 d cells were harvested from plates and stained for FACS analysis. Results from M-MDSC from **(A)** 4T1- or **(B)** RM1-tumor bearing mice. Shown are representative plots of the post-culture of Ly6C x Ly6G gate. Histograms were used to calculate the percent of $CD11b^+$ cells expressing Ly6G. Bars represent the mean + SEM for RM-1 data n=3, 4T1 n=4-5. In (A), bars with different letter superscripts are significantly different from one another ($p < 0.05$). In (B) the p value for the comparison is shown.

Downloaded from http://journals.aai.org/immunohorizons/article-supplement/237579/pdf/immunohorizon_2200079_supplemental_1/ by guest on 24 March 2025

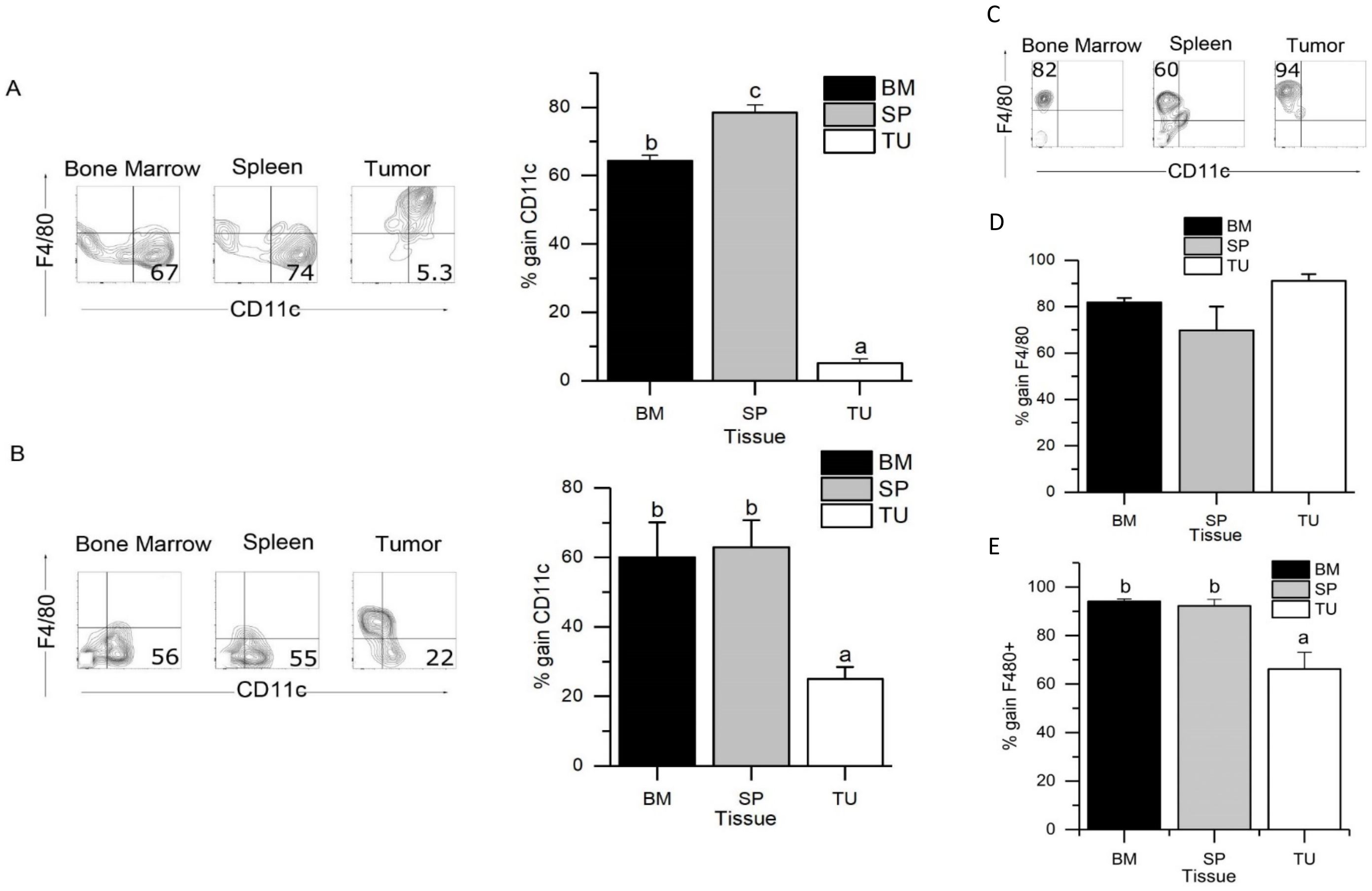

Figure S3: M-MDSC from RM-1 and 4T1 tumor models confirm findings TU M-MDSC are less able to become DC and that cells from all tissue are able to become macrophages. 4T-1 and RM-1 s.c. tumors were allowed to growth for 14d then M- subtype cells were isolated from bone marrow (BM), spleen (SP) and solid tumor (TU) and cultured for 3 days in RPMI-C with GM-CSF (10 ng/mL) and IL-4 (2 ng/mL). After 3d cells were harvested from plates and stained for FACS analysis. Results from M-MDSC from 4T-1(A) and RM-1 (B) tumor bearing mice (A-B, left) Shown are representative plots of post culture of CD11c x F4/80 gate BM, SP and TU. (A, *right*) Bar graph showing M-MDSC from BM and SP are significantly more able to gain CD11c compared to TU M-MDSC ($p<0.05$). (B, *right*) Bar graph showing M-MDSC from BM are significantly more able to gain CD11c compared to TU M-MDSC ($p<0.05$). Statistics were done using ANOVA on raw percentage data post Shapiro-Wilks normality assessment and Cook's D outlier test. Tumors from mice injected with RM-1 or 4T1 cells were allowed to grow for 14 d then M- subtype cells were isolated from bone marrow (BM), spleen (SP) and solid tumor (TU) and cultured for 3 days in RPMI-C with M-CSF (25 ng/mL). After 3d cells were harvested from plates and stained for FACS analysis. (C) Representative plots of post-culture of CD11c x F4/80 gate from RM-1 tumor model from BM, SP and TU. (D) Bar graph of RM-1 data. M-MDSC from BM, SP and, TU are not significantly different in their ability to gain F4/80 ($p<0.05$). Statistics were done using ANOVA on raw percentage data post Shapiro-Wilks normality assessment. (E) Bar graph of 4T1 data. M-MDSC from BM, SP and, TU were all able to gain F4/80 but the ability of TU M-MDSC was slightly lower than M-MDSC from the other two tissues ($p<0.05$).

Downloaded from http://journals.aai.org/immunohorizons/article-supplement/237579/pdf/immunohorizon_2200079_supplemental_1/ by guest on 24 March 2025

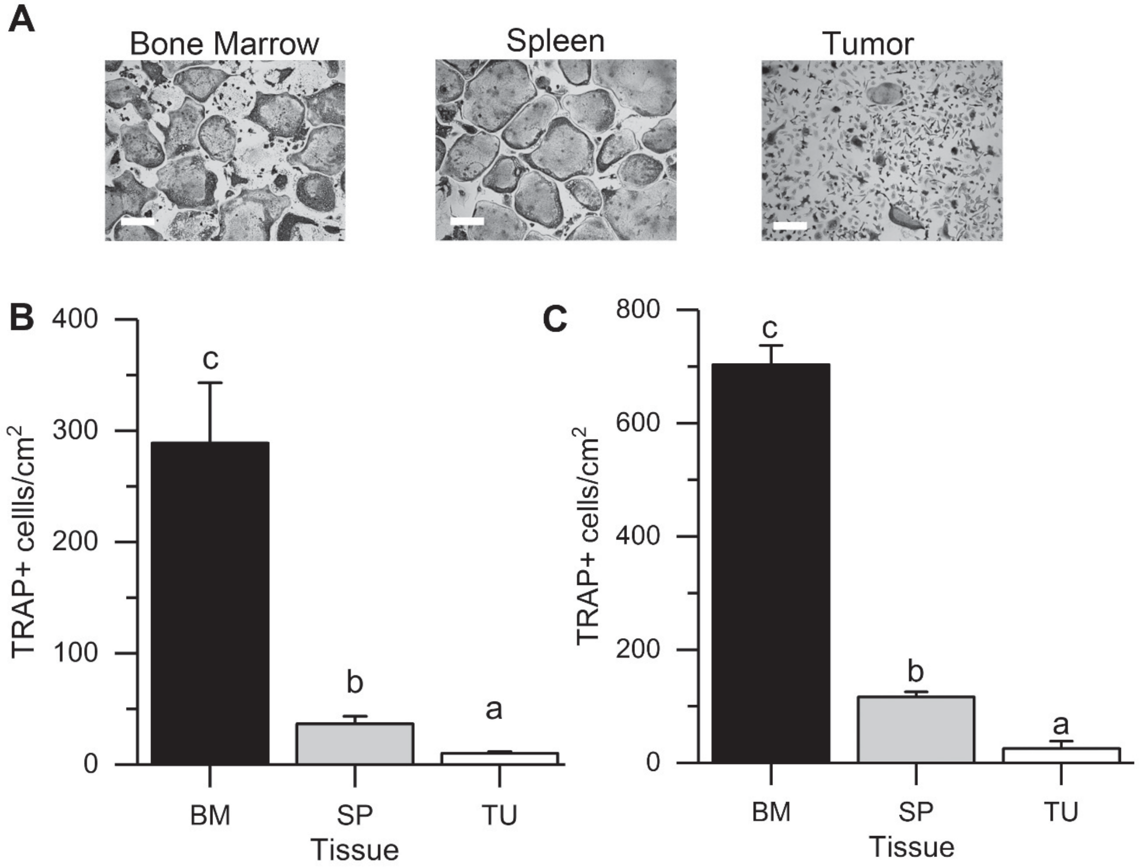

Figure S4: M-MDSC from RM1 and EL4 tumor models confirm findings TU M-MDSC are less able to become Osteoclasts. Tumors from mice injected with RM1 and EL4 cells were allowed to grow for 14 d then cells with M-MDSC markers were isolated from bone marrow (BM), spleen (SP) and solid tumor (TU) and cultured in M-CSF (50 ng/mL) and RANKL (100 ng/mL) for 8 d and then stained for TRAP+ cells and counted. (A) Representative micrographs of TRAP staining of M-MDSC from tumor-bearing mice post culture (40X). Bar represents 200 µm. (B) TRAP+ cells from RM1 derived tissue M-MDSC. (C) TRAP+ cells from EL4 derived tissue M-MDSC. Bars represent the mean + SEM (n=3). Bars with different letter superscript are significantly different from one another ($p<0.05$, Tukey's HSD).

Downloaded from http://journals.aai.org/immunohorizons/article-supplement/237579/pdf/immunohorizon_2200079_supplemental_1/ by guest on 24 March 2025
